# Evolution of networks in virtual reality: A scalable framework for studying social dynamics of small groups

**DOI:** 10.1371/journal.pone.0355470

**Published:** 2026-08-11

**Authors:** Rinseo Park, Mark Roman Miller, Eugy Han, Cyan DeVeaux, Jeremy N. Bailenson, Nilam Ram

**Affiliations:** 1 Department of Communication, Stanford University, Stanford, California, United States of America; 2 Department of Computer Science, Illinois Institute of Technology, Chicago, Illinois, United States of America; 3 Department of Media Production, Management, and Technology, University of Florida, Gainesville, Florida, United States of America; 4 Department of Psychology, Stanford University, Stanford, California, United States of America; Instituto Tecnologico Autonomo de Mexico, MEXICO

## Abstract

Immersive technologies provide new possibilities to study social dynamics. In this paper, we develop a methodological framework for identifying and describing the evolution of group networks in virtual reality (VR). Leveraging longitudinal data on participants’ interpersonal distances obtained in a collaborative virtual environment during a university course about VR we demonstrate how network methods can be applied and used to test propositions of social capital theory. Specifically, we use stochastic actor-oriented models (SAOMs) to explore the formation and change of social ties over time. Results showed that every additional connection to another group member reduced the likelihood of forming or maintaining a tie by less than half, whereas having a mutual connection (i.e., friend of friends) more than doubled the likelihood of tie formation or maintenance. This pattern supports our exploratory analysis that while students gradually became isolated in VR classrooms, their subgroups tended to persist. Also, participants were likely to change ties in ways that decreased imbalances in group identification and increased familiarity between dyad members, suggesting that social VR interactions are shaped by homophily. Overall, the substantive findings are indicative of bonding (ties inside the group), rather than bridging (ties outside the group), social capital. Methodologically, the integration of longitudinal network methods with VR tracking data opens new possibilities to learn about social dynamics.

## Introduction

Virtual reality (VR) technology facilitates social interaction in immersive collaborative virtual environments (CVEs), enabling people to engage in real-time communication within a shared virtual space via both verbal and nonverbal signals such as eye gaze and hand gestures [1–4]. In these environments, users can transcend their actual physical locations and form “virtual communities” that develop over time [5]. While recent studies have explored the evolution of psychological outcomes and interpersonal behaviors across multiple VR sessions [6–8], there remains a notable gap in research regarding the longitudinal formation of “relationship webs” in CVEs. This paper proposes a framework for using longitudinal network analysis based on the stochastic actor-oriented model (SAOM) [9–11] to investigate how social connections change over time in VR. Leveraging data collected during a 10-week university course focused on VR [6], we illustrate how longitudinal network analysis can be used to study the emergence, evolution, and decay of communities in CVEs, specifically examining mechanisms related to bridging and bonding social capital [12–14].

### Networks in virtual reality

Network formation is often conceptualized as a developmental process that emerges from the dynamics between constancy and change in group life [15,16]. Drawing on data such as classroom seating charts or online friendship networks, prior research describes how group composition or structural features evolve over time [17–20]. These studies highlight the key role peer-to-peer interactions play in driving these structural changes, increasing similarity among peers in a network through processes of selection, deselection, and influence [21]. For example, longitudinal network analysis across three repeated measurements of students’ friendship networks indicates that preadolescents tend to choose their friends based on similarity in popularity more than similarity in aggressive and prosocial behavior [22]. Extending from the studies done in traditional in-person classroom contexts, we illustrate how the study of changing social networks can be done in VR contexts. The passive and noninvasive tracking technologies embedded in CVEs allow for unobtrusive measurement of nonverbal cues, including body movements and interpersonal distances, that are indicators of social connection and underlying psychosocial processes [20,23–26]. Interpersonal distances and proxemics, for example, have been widely used as measures of social presence [27,28]. Leveraging this technology, we illustrate the possibility to reconstruct virtual communities from longitudinal data on micro-movements obtained in VR classrooms. As our theoretical framework, we integrate sociological and developmental views, with an emphasis on bridging and bonding social capital as mechanisms through which individuals establish, maintain, or dissolve social ties in CVEs.

#### Bridging and bonding social capital.

Social capital refers to resources embedded in social networks that individuals or groups can access through their social networks [13]. Coleman [12] argued that social capital enables social actors (individuals or groups) to choose partners for exchange of private or public goods. Individuals or groups can bond internally or bridge externally, and over time, these relational choices collectively reshape the broader social structure. As such, social capital is both inherent in social structure and influences the actions that shape group life, and thus is often regarded as a fundamental mechanism driving group formation and evolution; for an overview, see [13].

In brief, social capital often involves two types of network structures [14]. The first type, bridging social capital, manifests when individuals form ties outside their group, effectively expanding their social reach. These bridging ties typically manifest in broader and more loosely knit networks that facilitate exchanges across different communities or organizations. The second type, bonding social capital, manifests when individuals form ties inside the group. Often, these ties are reciprocal or transitive, such as connections formed among mutual acquaintances (i.e., friends of friends). These bonding ties typically exist within groups of homogeneous individuals, such as family members or close friends, leading to dense, cohesive networks that strengthen internal bonds and reinforce a shared identity. While both bridging and bonding social capital exist in social networks, bridging ties promote diversity and tolerance by linking individuals across group boundaries (e.g., strength of weak ties [29]), whereas bonding ties enhance trust and cohesion within groups. Generally, research suggests that a combination of both types is most effective in creating inclusive and accessible networks [30–32].

Empirical studies of in-person classroom interactions highlighting the importance of bonding structures that foster positive and inclusive peer relationships [33–35]. In contrast, research focused on online connections formed through social media platforms such as Facebook or Instagram generally demonstrates presence of interaction patterns related to bridging social capital [19,36–39]. However, this body of work consistently finds that social networks are shaped by homophily – the tendency for individuals to associate with others who are similar to themselves – which corresponds with bonding capital [30,32,40,41]. In VR contexts, studies have identified instances of homophily based on social characteristics such as gender and age [42], user behaviors like avatar personalization [43], and the role ingroup favoritism plays on attitude change [44]. Homophily also extends to nonverbal and behavioral patterns in VR environments. For example, verbal mimicry tends to decrease social distance and increase social attraction towards outgroup members [45]; as similarity of haptic patterns increases, participants both maintain closer interpersonal distances and report higher senses of belonging, social connection, and comfort toward virtual agents [46]. Building on this foundation, we illustrate the possibility of using longitudinal network analysis to study how bridging and bonding social capital manifest in virtual communities that form in CVEs.

### The present study/Motivating example

Social network analysis methods have been widely used in study of computer-mediated communication on social networking platforms such as Twitter [40] or Facebook [37], on computer-supported collaborative learning platforms [47], and in virtual environments such as friendship networks in Second Life [48]. In contrast, most of the research on immersive CVEs has focused on individual-level (e.g., self-avatar relationship) or dyad-level (e.g., interactions with other users or virtual agents) measures [1,7,42,43]. Although recent advances in VR technologies have enabled the capture of triadic and group-level features (e.g., gaze, motion synchrony) that may influence student outcomes [24,25], a substantial gap remains between the empirical analysis of the longitudinal data obtained in CVEs and Rheingold’s [5] original concept of virtual community as webs of personal relationships. In this paper, we develop and demonstrate how the longitudinal network analysis can be applied to such data to discover how communities form and evolve in VR.

**Fig 1:**
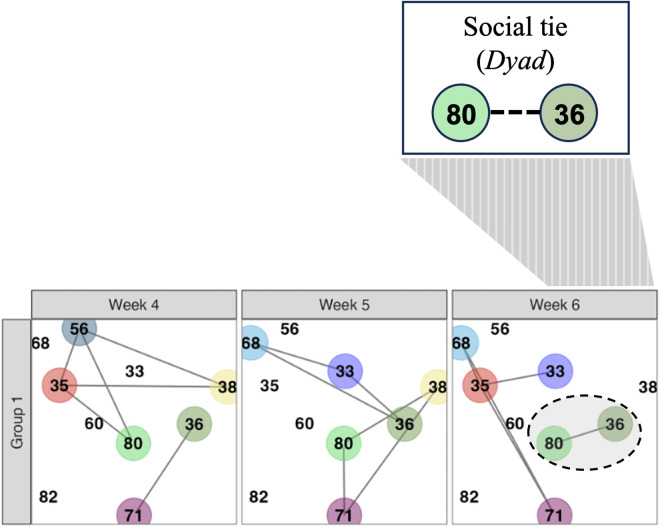
Schematic of SAOM analysis. The three panels in the bottom show the evolution of ties among the group (Group 1) across three observations (Weeks 4-6). A particular social tie between two actors #80 and #36 in Week 6 is highlighted to illustrate that SAOMs model single-tie-changes.

The three panels in Fig 1 show the evolution of ties among a group of six students observed on multiple occasions in a CVE classroom. Consider the particular social tie highlighted on the right, that is, a tie (edge) between two actors (circles, #80 and #36). Our aim is to determine if and how the connection between these two participants (and all the other connections in the network) emerged or changed over time.

**Fig 2:**
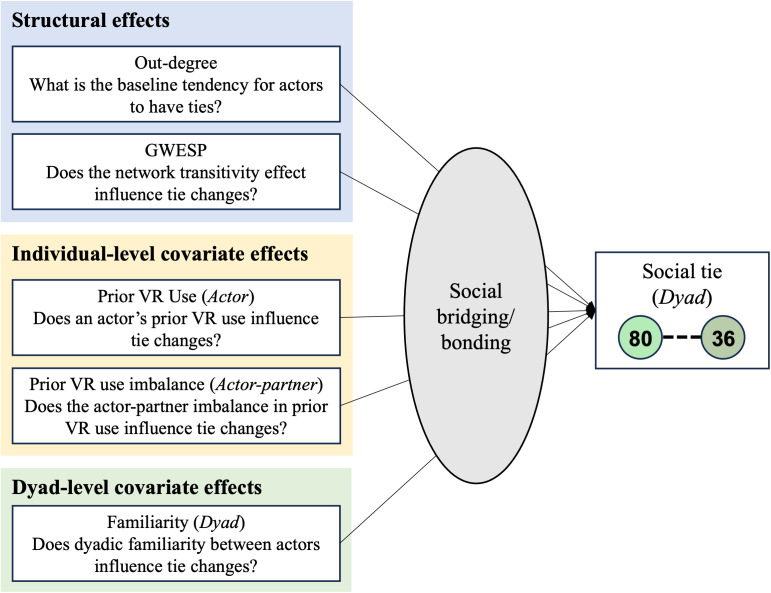
Types of effects and the corresponding research questions in SAOM analysis. Three different types of effects (shown as boxes on the left) included to examine how bridging and bonding processes (shown as an ellipse in the middle) influence the formation or change of social ties (shown as a box on the right; an edge between two actors in a given network): structural network features that are indicative of group activity (blue), individual-level covariates (yellow; actor- or actor-partner effects), and dyad-level covariates (green; dyadic effects).

Fig 2 outlines the type of research question motivating our foray into longitudinal network analysis of CVE data and emergence of community in VR. As illustrated, we examine how bridging and bonding processes (shown as an ellipse in the middle) manifest using the three different types of effects (shown as boxes on the left) – structural effects (blue), individual-level covariates (yellow), and dyad-level covariates (green) – that may be influencing the formation, maintenance, or dissolution of proximity-based social ties. For the covariate effects, each box corresponds to actor, actor-partner, or dyadic effect and addresses a specific research question. Using this example, we forward longitudinal social network analysis as a scalable framework for studying social dynamics of small groups in CVEs.

## Stochastic actor-oriented model

### Overview

Social network analysis allows researchers to examine the structure of social groups and the mechanisms underlying group dynamics [49–51]. Stochastic actor-oriented models (SAOMs) [9] can be used to test hypotheses about and explore how social ties form and change over time. While exponential random graph models (ERGMs) are commonly used for modeling tie existence in cross-sectional network data [11,48,52,53], SAOMs are specifically oriented toward modeling the evolution of longitudinal networks [10,54] (see [11] for an overview of related models such as temporal ERGM). SAOMs focus on the role of individuals in forming (or changing) social ties over time. Individuals are treated as *social actors* having the potential to change their outgoing ties, and the observed network dynamics are the result of a sequence of choices by these actors. Tie changes are conceptualized as a *stochastic* process in which the likelihood of forming or dissolving a social tie is determined by a collection of effects that include network structures and actor- or dyad-level covariates. Particularly relevant for our substantive inquiry, the construction of the SAOM aligns with the self-organizing nature of group interactions in classrooms – see [35] for an overview of traditional classroom settings – and the view of social ties as pathways for accessing and leveraging social capital. Accordingly, we apply SAOMs to examine how bridging and bonding forms of social capital are embedded in, and evolve through, VR group interactions.

### Notation

In SAOM analyses, temporal changes in social ties are modeled as a continuous-time Markov process where actors form, maintain, or dissolve ties in a probabilistic way that is informed by the current network structure and individual behavior [10,11,55]. A social network of interest that is composed of *N* actors (e.g., course participants meeting in a CVE) is generally denoted and modeled as a (un)directed graph represented by an N×N adjacency matrix 𝐱, where $𝐱=(x_{ij})$ indicates the presence or absence of social ties between actors *i* and *j*. For simplicity of presentation, we assume here that $x_{ij}$ is a binary tie variable in a non-directed network (i.e., $x_{ij}=x_{ji}$) where $x_{ij}=1$ indicates the presence of a tie between actor *i* and actor *j*, and $x_{ij}=0$ indicates the absence of a tie; for other tie values that are directed or ordered, see [11]. Self-ties are not permitted, so the diagonal elements of the matrix where *i* = *j* are all set to zero. The longitudinal model assumes that the network 𝐱(t) evolves in continuous time, that all the actors in the network get opportunities to change their ties at discrete time points along the way (ministeps), and that the network is observed at just a few of those discrete time points, ($t_{1},\cdots ,t_{2},\cdots ,t_{3},\cdots ,t_{M}$) where the ellipses indicate unobserved ministeps between the *M* points where actual observations of the network are obtained.

### Model definition

Two main functions are used to formulate the SAOM: a *rate function* that governs the frequency with which actors have opportunities to change their ties (ministeps), and an *objective function* that describes how particular network-, individual-, and dyad-level characteristics govern the choices about which ties to change (see S1 Text for details).

Typically, the rate function is defined by Eq (1), where $\rho_{m}$ is the rate parameter in a time interval $t_{m}<t\leq t_{m+1}$ (m=1,⋯,M−1) for all the actors in the network. When actor *i* gets the opportunity to change, this actor can engage with only one tie variable $x_{ij}$ (i.e., they are only allowed to change up to one tie variable at each ministep).


$$\begin{array}{c} \lambda_{i}\left(𝐱(t)\right)=\rho_{m} \end{array}$$ (1)

The objective function is defined by Eq (2), where ${𝐱}^{\left(\pm ij\right)}$ denotes a potential state of the network after toggling the specific single tie $x_{ij}$ and $s_{ik}({𝐱}^{\left(\pm ij\right)})$ denotes actor *i*’s evaluation of the *k*-th *effect* (k=1,⋯,K) for that potential state.


$$\begin{array}{c} f_{i}({𝐱}^{\left(\pm ij\right)}\;|\;\beta )=\sum_{k=1}^{K}\beta_{k}s_{ik}({𝐱}^{\left(\pm ij\right)}) \end{array}$$ (2)

The $\beta_{k}$ parameters in $\beta =(\beta_{1},\beta_{2},\cdots ,\beta_{K})$ describe the extent to which each of the specific effects contributes to actors’ choices about which ties to toggle, and thus to the evolution of the network over time. As listed in Table 1 and illustrated by the three boxes on the left of Fig 2, these effects typically fall into three categories: (1) structural effects, (2) individual-level covariate effects, and (3) dyad-level covariate effects.

**Table 1:** Structural, individual-level covariate, and dyad-level covariate effects included in SAOMs.

Effect	Equation	Bridging	Bonding
**Structural effects**			
Out-degree	$s_{i,\text{OD}}\left(𝐱\right)=\sum_{j}x_{ij}$	–	–
Transitive triads	$s_{i,\text{transTriads}}\left(𝐱\right)=\sum_{j<k}x_{ij}x_{jk}x_{ki}$	$\beta_{\text{transTriads}}<0$	$\beta_{\text{transTriads}}>0$
GWESP	$s_{i,\text{GWESP}}\left(𝐱\right)=\sum_{j}x_{ij}e^{\alpha }\{1-{\left(1-e^{-\alpha }\right)}^{\Sigma_{h}x_{ih}x_{hj}}\}$	$\beta_{\text{GWESP}}<0$	$\beta_{\text{GWESP}}>0$
**Individual-level covariate effects**			
Time (actor)	$s_{i,\text{time}}\left(𝐱\right)=\sum_{j}x_{ij}{\text{time}}_{i}$	–	–
Prior VR use (actor)	$s_{i,\text{priorVR}}\left(𝐱\right)=\sum_{j}x_{ij}{\text{priorVR}}_{i}$	–	–
Prior acquaintance (actor)	$s_{i,\text{priorAcq}}\left(𝐱\right)=\sum_{j}x_{ij}{\text{priorAcq}}_{i}$	–	–
Group identification (actor)	$s_{i,\text{groupIdn}}\left(𝐱\right)=\sum_{j}x_{ij}{\text{groupIdn}}_{i}$	–	–
Prior VR use imbalance	$s_{i,\text{priorVR.Imb}}\left(𝐱\right)=\sum_{j}x_{ij}|{\text{priorVR}}_{i}-{\text{priorVR}}_{j}|$	$\beta_{\text{priorVR.Imb}}>0$	$\beta_{\text{priorVR.Imb}}<0$
(abs. difference; actor-partner)			
Prior acquaintance imbalance	$s_{i,\text{priorAcq.Imb}}\left(𝐱\right)=\sum_{j}x_{ij}|{\text{priorAcq}}_{i}-{\text{priorAcq}}_{j}|$	$\beta_{\text{priorAcq.Imb}}>0$	$\beta_{\text{priorAcq.Imb}}<0$
(abs. difference; actor-partner)			
Group identification imbalance	$s_{i,\text{groupIdn.Imb}}\left(𝐱\right)=\sum_{j}x_{ij}|{\text{groupIdn}}_{i}-{\text{groupIdn}}_{j}|$	$\beta_{\text{groupIdn.Imb}}>0$	$\beta_{\text{groupIdn.Imb}}<0$
(abs. difference; actor-partner)			
Same gender (actor-partner)	$s_{i,\text{gender}}\left(𝐱\right)=\sum_{j}x_{ij}I\{{\text{gender}}_{i}={\text{gender}}_{j}\}$	$\beta_{\text{gender}}<0$	$\beta_{\text{gender}}>0$
Same ethnicity (actor-partner)	$s_{i,\text{ethnicity}}\left(𝐱\right)=\sum_{j}x_{ij}I\{{\text{ethnicity}}_{i}={\text{ethnicity}}_{j}\}$	$\beta_{\text{ethnicity}}<0$	$\beta_{\text{ethnicity}}>0$
**Dyad-level covariate effects**			
Familiarity (dyad)	$s_{i,\text{familiarity}}\left(𝐱\right)=\sum_{j}x_{ij}{\text{familiarity}}_{ij}$	$\beta_{\text{familiarity}}<0$	$\beta_{\text{familiarity}}>0$
Friendship (dyad)	$s_{i,\text{friendship}}\left(𝐱\right)=\sum_{j}x_{ij}{\text{friendship}}_{ij}$	$\beta_{\text{friendship}}<0$	$\beta_{\text{friendship}}>0$

*i* denotes a focal actor, *j* denotes another actor in a dyad *i*, *j*, *h* denotes the third actor in a triad *i*, *j*, *h*. In the equation for GWESP (geometrically weighted edgewise shared partners), α is a tuning parameter which is typically fixed at ln(2).

***Structural effects*** are based on the topology of the network, capturing how actors’ placement and role in the surrounding network influence their choices about which ties to form, maintain, and dissolve. For example, out-degree indicates the tendency of actors to form ties with other actors. As shown for *OD* in Table 1, out-degree is quantified as the total number of out-going ties. As a complementary feature of the network, reciprocity indicates the tendency of actors to form mutual ties. For example, reciprocity can be quantified as the total number of ties where person *i* is connected to person *j* and person *j* is connected to person *i*. In the case of non-directed ties where the connections are always mutual, the quantifications of out-degree and reciprocity are equal and thus not included in Table 1. A third core aspect of network topology is transitivity, the tendency for actors to close triads and form clusters shown by the number of transitive patterns in actor *i*’s relation (*transTriads*) that quantifies the extent that “a person’s friends are also friends with each other” and the geometrically weighted edgewise shared partners effect (*GWESP*) that uses a concave logarithmic function to capture nonlinear tendencies in network closure [56]. Beyond these basic quantifications, a variety of structural effects that may contribute to individuals’ decisions about what ties to change at each ministep have been elaborated [51,57]. In particular, degree-related effects can be useful for network analysis where degrees are theoretically important or represent social status. For example, degree activity plus popularity effect (*degPlus*, [57]) is defined specifically for non-directed networks. In the analysis, we attempted to include this effect at both raw and square root versions and neither converged due to small network sizes. As will be detailed below, the $\beta_{k}$ parameters associated with each of the structural effects $s_{ik}$ indicate how the different aspects of the network topology influence actors’ decisions about tie changes.

***Individual-level covariate effects*** are those that involve individual difference measures, including demographic or behavioral measures derived from a given social context. For example, in the current study, we examine how time, participants’ prior VR use, their prior acquaintance, and their tendency for group identification influence the formation, maintenance, and dissolution of ties within VR networks. As shown for *time*, *priorVR*, *priorAcq*, and *groupIdn* in Table 1, those measures can be integrated into the objective function via summation across existing ties. While these **actor effects** capture whether and how individuals’ characteristics are related to tendency for tie formation, **actor-partner effects** facilitate examination of, for example, whether beginner and expert VR users tend to form ties with others of similar or different level of prior experience or whether participants of the same demographic background are more likely to interact with each other. These effects include imbalance effects – each calculated as the sum of absolute differences in prior VR use (*priorVR.Imb*), prior acquaintance (*priorAcq.Imb*), and group identification (*groupIdn.Imb*) – as well as same-covariate effects for gender (*gender*) and ethnicity (*ethnicity*). Together, the $\beta_{k}$ parameters associated with each of the individual-level covariate effects indicate how individual differences influence actors’ decisions about tie changes.

***Dyad-level covariate effects*** are those that involve individuals’ perceptions of their dyadic relationships. In the current study, we examine how study participants’ familiarity and friendship with each other person in their group influence their decisions to form, maintain, and dissolve ties over time in the CVE. As shown for *familiarity* and *friendship* in Table 1, these effects are integrated into the objective function via summation across existing ties. Notably, different from the individual-level covariate effects, these effects are not necessarily symmetric, as person *i* may consider themselves more familiar with person *j* than person *j* considers themselves familiar with person *i*.

Combining together the structural, individual-level covariate, and dyad-level covariate effects, selection of an $x_{ij}$ that might be changed in a ministep by actor *i* (relative to all other possibilities $x_{ih}$) is modeled by a conditional multinomial logistic model of the form Eq (3). Of particular interest for hypothesis testing are the $\beta_{k}$ parameters indicating the direction and extent to which each of the structural and covariate effects $s_{ik}$ is associated with tie changes.


$$\begin{array}{c} p_{i}\left({𝐱}^{\left(\pm ij\right)}\;|\;\beta \right)=\frac{\exp\left(f_{i}({𝐱}^{\left(\pm ij\right)}\;|\;\beta )\right)}{\sum_{h}\exp\left(f_{i}({𝐱}^{\left(\pm ih\right)}\;|\;\beta )\right)}=\frac{\exp\left(\sum_{k=1}^{K}\beta_{k}s_{ik}({𝐱}^{\left(\pm ij\right)})\right)}{\sum_{h}\exp\left(\sum_{k=1}^{K}\beta_{k}s_{ik}({𝐱}^{\left(\pm ih\right)})\right)} \end{array}$$ (3)

### Model estimation and selection

The parameters in the SAOM are usually estimated using the Method of Moments (MoM) wherein the system of estimating equations is based on the statistics corresponding to the rate function and objective function, respectively. From the rate function Eq (1), the vector of period-specific rates $\rho =(\rho_{1},\rho_{2},\cdots ,\rho_{M-1})$ is estimated using statistics derived from the total number of tie changes that were observed to have accumulated in the periods between successive observations $t_{m}$ and $t_{m+1}$ of the network (m=1,⋯,M−1). These rates are calibrated such that the expected amount of change over the time interval matches the observed Hamming distance [10]. In practice, larger rate parameters may indicate model misspecification, providing sufficiently high change opportunities for the model to compensate self-canceling ties or to facilitate unlikely but relevant choices. From the objective function Eq (2), the vector of effect-specific coefficients $\beta =(\beta_{1},\beta_{2},\cdots ,\beta_{K})$ is estimated using statistics derived from the sum of each effect $s_{ik}({𝐱}^{\left(\pm ij\right)})$ over all actors i=1,⋯,N across *M* time points, so that higher values of $\beta_{k}$ will lead to networks ${𝐱}^{\left(\pm ij\right)}$ with higher values of $s_{ik}({𝐱}^{\left(\pm ij\right)})$. Thus, in our case, the vector of parameter values (ρ,β) is determined by solving two first moment equations. Because the model is too complex to obtain closed-form solutions, stochastic simulation algorithms are used for estimation, such that simulated data matches the observations in expectations on the target statistics. In practice, the R package “RSiena” implements a three-phase algorithm: the first phase computes the sensitivity of the expected statistics to parameters, the second phase uses multivariate Robbins-Monro updates based on simulated networks to adjust differences between expected and observed statistics, and the third phase simulates networks using the final parameter values and calculates standard errors. Following model estimation, it is necessary to check that convergence has been achieved. A threshold of 0.25 is commonly used for the overall maximum convergence ratio, and a threshold of 0.1 for the absolute value of the individual *t*-statistics. Troubleshooting steps for estimation difficulties (e.g., deviations are above the threshold) can be found in the RSiena manual [57]. The estimation algorithm is by default conditional on the observed number of changes – also known as conditional MoM – which is known to be more stable and efficient [57]. For other estimation methods, including likelihood-based and Bayesian methods, please refer to [11].

After estimation, model selection is typically conducted using multi-parameter Wald tests to assess the null hypothesis that a model with one or more parameters constrained equal to zero fits the data as well as a model where those parameters are unconstrained. In more exploratory settings, forward model selection procedures based on Mahalanobis distance-based Monte Carlo (MDMC) tests may be useful for identifying the specific structural effects to include as predictors in the objective function [58]. Once a good fit between model and data is achieved, hypothesis testing and interpretation of model parameters can follow.

### Interpretation of model parameters

Following the general set-up shown in Fig 2, we highlight how the SAOM can be used to test whether the set of structural and individual- and dyad-level covariate effects shown on the left side of the figure is related to evolution of ties among participants in VR classrooms shown on the right side of the figure. In the rate function, the rate parameters ρ indicate how many times individuals might get the opportunity to change their ties during each period (in multigroup analyses, they are also group-specific) [59]. Because these parameters are mostly about how the simulations are run in the estimation procedure and are not tied to substantive hypotheses, they are generally treated as nuisance parameters, especially when the rate function does not depend on structural or covariate effects [55,59–61]. The rate function can be useful for extensions such as modeling the co-evolution of networks and behavior or diffusion of innovation [11,62]. In contrast, the effect parameters β in the objective function are often core to the substantive hypotheses and inferences about how and why the networks change over time. Generally, $\beta_{k}$ parameters that are significantly different than zero are interpreted as in typical logistic regressions: a 1-unit increase in the structural or covariate effect, $s_{ik}$, is associated with an increase in the odds ratio of tie change by $\exp(\beta_{k})$. To illustrate, we outline how a few exemplar effects examined in this study might proceed in relation to hypotheses about social capital theory.

#### Interpretation of structural effects.

First, the out-degree effect is interpreted as the baseline tendency for actors to have ties. Specifically, a positive coefficient for the out-degree effect indicates a stronger tendency to form or maintain ties, as the objective function value increases with an additional tie in the potential state ${𝐱}^{\left(\pm ij\right)}$. For illustration, consider a network where the tie of interest is currently absent ($x_{ij}=0$), with two potential states: (a) ${𝐱}^{\left(\pm ij\right)}$ has an additional tie $x_{ij}=0\to 1$ vs. (b) ${𝐱}^{\left(\pm ij\right)}$ is the status quo. In this case, a one-unit increase in out-degree effect with parameter $\beta_{\text{OD}}=0.5$ increases the log-odds of forming a new tie (a) over the status quo (b) by 0.5, which corresponds to an odds ratio $\exp(\beta_{\text{OD}})=1.65$. That is, the likelihood of forming a new tie increases by 1.65 times. The out-degree effect is generally considered as an intercept rather than a substantive finding; its value is determined by network size and density and can be affected by the inclusion of other degree-related effects. Second, the GWESP effect is interpreted as indicating bridging social capital when $\beta_{\text{GWESP}}<0$ and bonding social capital when $\beta_{\text{GWESP}}>0$ (as well as reciprocity and transitivity effects in Table 1). A positive GWESP coefficient indicates a stronger tendency to connect with friends of friends.

#### Interpretation of individual-level covariate effects.

This interpretation also extends to covariate effects, especially those representing actor-partner effects such as imbalance or same-covariate effects. On the one hand, imbalance effects – measuring dissimilarity between focal actor and its partners – are interpreted as indicating bridging social capital when $\beta_{\text{Imb}}>0$ and bonding social capital when $\beta_{\text{Imb}}<0$. For example, a negative coefficient for prior VR use imbalance effect ($\beta_{\text{priorVR.Imb}}<0$) indicates that participants tend to form and maintain ties that can decrease imbalance – as manifestations of bonding capital (connect with similar others, disconnect with dissimilar others). On the other hand, same-covariate effects are interpreted as indicating bonding social capital when $\beta_{\text{gender}}>0$ or $\beta_{\text{ethnicity}}>0$ and bridging social capital when $\beta_{\text{gender}}<0$ or $\beta_{\text{ethnicity}}<0$. Although not directly related to social capital theory, interpretation of actor effects can be useful to test hypotheses related to how individual characteristics affect VR interaction dynamics. For example, a positive coefficient for prior VR use indicates that ties are more likely to be created or maintained between participants with prior VR experience.

#### Interpretation of dyad-level covariate effects.

Dyad-level covariate effects can be interpreted in parallel with actor-partner effects at the individual level. Especially, dyadic similarity effects – measuring similarity between dyad members like familiarity or friendship – are interpreted as indicating bonding social capital when $\beta_{\text{familiarity}}>0$ or $\beta_{\text{friendship}}>0$ and bridging social capital when $\beta_{\text{familiarity}}<0$ or $\beta_{\text{friendship}}<0$. For example, a positive familiarity coefficient implies a preference for ties that can increase familiarity – as manifestations of bonding capital (connect with familiar others, disconnect with unfamiliar others).

Taken together, these parameters govern how bridging or bonding social capital in VR networks. First, bridging processes manifest when individuals tend to associate with (a) popular others who can maximize their social reach, or among (b) heterogeneous populations with different backgrounds. In other words, bridging social capital is identified by (a) high tendency to form ties with popular others and (b) low similarity between individual profiles. In the current analysis, bridging social capital is supported when dissimilarity-based covariate effects (imbalance effects in Table 1) are positive, while GWESP and similarity-based covariate effects (same-covariate and dyadic effects in Table 1) are negative. Second, bonding processes manifest when individuals tend to associate with (c) familiar others from ingroup relationships (e.g., family members, close friends), or among (d) homogeneous populations with similar backgrounds. In other words, bonding social capital is identified by (c) high network closure (reciprocity, transitivity) and (d) high similarity between individual profiles. Thus, in our analysis, bonding social capital is supported when the GWESP and similarity-based covariate effects (i.e., same gender/ethnicity, dyadic familiarity/friendship) are positive, while dissimilarity-based covariate effects (e.g., prior VR use imbalance) are negative.

## Materials and methods: Longitudinal VR data

Data for our illustrative example are drawn from a large-scale, longitudinal study of interaction behavior within CVEs, accessed through standalone, untethered headsets [6]. During a 10-week university course about VR, participants were provided with an Oculus Quest 2 VR headset to attend weekly small-group (sizes ranging from 9 to 14) discussion sessions on the ENGAGE platform for eight weeks. Teaching staff members provided technical support, assisted with small-group activities within the virtual environment, and facilitated weekly data collection. Previous analyses of these data showed that participants’ perceptions of and behaviors within their virtual experience shifted systematically across weeks, and that prior VR use, having prior acquaintances with other participants, and levels of group identification were associated with individual differences in people’s experiences in virtual environments such as presence and realism, as well as group outcomes such as synchrony and entitativity [6,63]. In the current study, we focused on a 5-week subset of the data to illustrate how longitudinal social network analysis can be used to study small group dynamics in virtual worlds. During this five-week period, participants in each group were instructed to navigate a CVE on the ENGAGE platform and freely position themselves to engage in small-group discussion activities. Study details relevant to this methodological illustration are given below. The data underlying this article cannot be shared publicly due to the privacy of individuals that participated in the study. The data will be shared on reasonable request to the Virtual Human Interaction Lab (VHIL) at Stanford University (https://vhil.stanford.edu/).

### Ethics statement

All procedures were approved by the Stanford University Institutional Review Board (eProtocol 61257) and additionally reviewed by the Stanford University Student Data Oversight Committee (SDOC). All participants provided written informed consent at the beginning of the study. While all students who enrolled in the 10-week course took part in all the VR activities, only those who consented to participate contributed data for analysis. To minimize any risk of coercion, the consent process and data collection were overseen by a third party. Participants were reminded at the beginning of every session that they were being recorded. The study did not include minors.

### Participants

Our analysis focuses on 81 participants enrolled in a university course during the Summer quarter of 2021 (June 21 to August 27, 2021). Participants were included if they consented to the study and attended at least five of the eight weekly VR sessions. The final sample ranged in age from 18 to 58 years (*M* = 22.26, *SD* = 5.19); further demographic information (gender, ethnicity) is provided in the Measures section.

### Procedure

During the course, participants were organized into eight groups to take part in weekly VR sessions (9–14 persons per group; *M* = 12.63, *SD* = 1.77). In the weekly VR sessions, participants engaged in a variety of individual, small-group, and full-group learning tasks for approximately 30 minutes. In Week 1, participants acclimated to the VR headset and ENGAGE platform. During Weeks 2 and 3, participants were instructed to stand in a circle for full-group discussions. During Weeks 4–8, participants in each group were encouraged to engage in small-group discussions within a large open space where they could walk or transport freely for small-group discussion. Comprehensive information on weekly discussion activities and discussion topics is given in Table 1 of [6]. All activity and events occurring in the CVE were tracked via “.myrec” recordings which were outputted by the ENGAGE platform, which capture all the nonverbal behaviors from participants’ avatars (i.e., virtual body) and physical bodies (e.g., head and hands), audio, and the location and movement of all other objects in 3D space. The nonverbal behaviors of specific interest here were captured through positional (x: left/right, y: up/down, z: forward/backward) and rotational (x: pitch, y: yaw, z: roll) data for the avatars (tracked as the root, or the center of the avatar, within the virtual environment) and the participants’ physical head and hands (tracked by the VR headset and right and left controllers). At the start of the study (pre-test), during and after each weekly VR session, and at the end of the course (post-test), participants completed Qualtrics questionnaires reporting their attitudes toward the VR experience (e.g., presence, enjoyment), their familiarity and friendship with group members, and overall VR experience. For the network analysis presented here, we focused on interpersonal distances derived from tracking data obtained in Weeks 4–8, which were focused on activities where participants were instructed to freely position themselves and discuss in small groups with other participants, and selected pre-test and post-test questionnaire data that were collected at the beginning and end of the course.

### Measures

#### Interpersonal distance.

Interpersonal distances in virtual space ($distance_{ijt}$) were calculated for each pair of participants (*i*, *j*) in each weekly session (*t*) as the mode of their distances across all 1/3-me*t*er spatial bins and 1/30-second temporal epochs (30 Hz) of every 30-minute VR session. Specifically, each combination of spatial and temporal binning generates a distribution of distance values between each pair participants (*i*, *j*), which were then summarized as one value (e.g., minimum) [25] to represent the interpersonal distance at that weekly session (*t*). To represent the distance a*t* which participants spent the most time, we used the mode of their pairwise distances during each 30-minute session. As will be described later in the Stage 1 of Data Analysis section, the social ties within each group at each weekly session were derived from these interpersonal distances.

Building on the existing literature on proxemics, which used in-VR interpersonal distances as measures of social interaction and the underlying psychological processes [27,28], our use of mode as the summary statistic for each weekly session provides both substantive and empirical justifications for the SAOM method. Substantively, while close-distance interaction can also occur for reasons unrelated to meaningful social interaction (e.g., standing next to someone while listening to the teaching team, passing by while moving across the classroom), especially for short intervals such as 1/30-second epochs, the mode of distances for a given pair of participants across all epochs for each 30-minute session (=30×60×30 epochs) provides a reasonable proxy for their latent social relationship underlying VR interaction. Empirically, the use of the mode addresses the concern of physical dependence. At a given single time point (e.g., 1/30-second epoch), a decrease in one distance value (e.g., staying closer to one person; forming a tie) can accompany decreases in other distance values (e.g., getting closer to other people nearby; forming multiple ties), potentially violating the single-tie-change assumption [64] fundamental to network-based approaches (e.g., SAOM, ERGM; see the Methods section). However, this dependence no longer holds at the aggregate 30-minute session level.

#### Time-invariant individual difference measures.

Five time-invariant individual-level covariates were included in the analysis. First, three variables measuring participants’ prior VR use and group relationships were included:

**Prior VR use:** As part of a larger pre-test questionnaire, participants indicated how many times they had experienced VR (*n*_0_ = 42, *n*_1_ = 6, *n*_2_ = 6, *n*_3_ = 7, *n*_4+_ = 20). For simplicity, responses were recoded to obtain a simple binary prior VR use variable: = 1 if the participant had ever experienced VR (*n*_1+_ = 39; 48%) and =0 otherwise (*n*_0_ = 42; 52%). The remaining one participant had missing data for this measure.

**Prior acquaintance with group members:** Participants also indicated how many group members they were familiar with prior to the course (i.e., “How many people in this discussion section were you familiar prior to this quarter? (e.g., 0, 1, 2, 3)”, *n*_0_ = 38, *n*_1_ = 13, *n*_2_ = 12, *n*_3_ = 1, *n*_4_ = 2, *n*_5_ = 2, did not respond = 13). As certain participants had more relationships prior to the study (e.g., student athletes), this individual-level measure helps account for these individual differences. For simplicity, responses were recoded to obtain a simple binary prior acquaintance variable: = 1 if the participant had any prior acquaintance within their assigned discussion groups (*n*_1+_ = 30; 37%) and =0 otherwise (*n*_0_ = 38; 47%). The remaining 13 participants had missing data for this measure.

**Group identification:** Participants’ general tendency to identify with groups was measured in the pre-test questionnaire using eight items adapted from an in-group identification scale and an organizational identification scale [65,66]. Participants rated the extent to which they identify with the groups they belong to (e.g., organizations at home, clubs in school, sports teams) using a 7-point Likert scale (1 = Strongly disagree, 7 = Strongly agree). Examples of items include “I feel a bond with my group,” “People in my group have a lot in common with each other,” and “When I talk about my group, I usually say ‘we’ rather than ‘they.’” Each individual’s group identification score was calculated as the average of their responses to the eight items (*M* = 5.35, *SD* = 0.97; Cronbach’s α = .89). For interpretability, this measure was group-mean centered so that positive values indicate greater than average group identification.

Second, two variables measuring participants’ gender and ethnicity were included:

**Gender:** As part of the pre-test questionnaire, participants indicated their gender by selecting from multiple categories (Female = 30, Male = 47, Other = 2, declined to respond = 1, did not respond = 1).

**Ethnicity:** Participants also reported their ethnicity in the pre-test questionnaire by selecting from multiple categories (African American or Black = 11, Asian or Asian American = 30, Hispanic or Latinx = 9, Middle Eastern = 1, White = 21, more than one race = 5, declined to respond = 3, did not respond = 1).

#### Time-invariant dyadic relationship measures.

Two time-invariant dyad-level covariates were included in the analysis:

**Familiarity:** At the post-test questionnaire, participants rated how familiar they were with each member in their group (i.e., in a group of *N* individuals, each person provides N−1 familiarity ratings). Participants were shown the name of each group member and asked, “How well do you know this member?” using a 5-point Likert scale (1 = Not familiar at all, 5 = Extremely familiar; except “Self/NA”). Familiarity ratings were not reciprocal within each pair of participants (i.e., A’s familiarity with B could differ from B’s familiarity with A), resulting in a nonsymmetric (directed) matrix of familiarity scores (*M* = 2.13, *SD* = 1.16). This matrix was mean centered, so that positive values indicate greater familiarity than the average of all dyads.

**Friendship:** Participants’ level of friendship with each member in their group was measured at the post-test questionnaire. Participants were shown the name of each group member and asked to indicate their agreement with the statement, “I would be excited to get to know this member better,” using a 7-point Likert scale (1 = Strongly disagree, 7 = Strongly agree; except “Self/NA”). As with familiarity, friendship ratings were not reciprocal within each pair of participants (i.e., A’s friendship with B could differ from B’s friendship with A), resulting in a nonsymmetric (directed) matrix of friendship scores (*M* = 4.99, *SD* = 1.27). This matrix was mean centered, so that positive values indicate greater friendship than the average of all dyads.

#### Time metric.

Drawn from the five weeks (Weeks 4−8) of the study, time was indicated by the week variable after mean centering (range of *t* = −2–2) so that *t* = 0 indicates Week 6.

## Data analysis

We demonstrate how SAOM methods and newly available longitudinal network data – obtained during a university course about VR – can be used to identify and examine group dynamics: How do individuals join, stay connected to, and leave networks established in virtual classrooms? The SAOM framework we introduce here is invoked in two stages. In the first stage of analysis, we define and design the longitudinal network data, specifically the multigroup structure, the binary tie variable, and the logic of tie changes. In the second stage of analysis, a series of SAOM models is used to test whether the set of hypothesized structural, individual-level covariate, and dyad-level covariate effects influence tie formation and change.

### Stage 1: Design of longitudinal network

The first stage consists of three considerations: structuring the multigroup network data, designing the binary tie variable, and specifying the logic of tie changes.

#### Multigroup network.

In the dataset, five weekly behavioral and attitudinal measures were nested within 81 individuals, who were in turn nested within eight groups. This resulted in a repeated measures data structure with five weekly networks nested within eight groups. Accordingly, we conducted a multigroup SAOM analysis that offers an introductory approach for studying longitudinal dynamics within VR classroom networks [57,60,67]. The multigroup analysis separates the set of longitudinal networks by group – treating group as a categorical variable. The parameters in the rate function are group-specific (i.e., each of the eight groups has its own period-specific rates), but that the parameters in the objective function are equal across groups. We note that this is a set of strong homogeneity assumptions, and for model extensions such as meta-analysis or multilevel analysis that allow parameters in the objective function to be group-specific, see [59].

#### Binary tie variable.

SAOMs operate on binary networks [56,57], where ties between actors have only one of two states: existent (1) or non-existent (0). In our analysis, the binary networks for each of the eight groups were derived from continuous, non-directional measures of interpersonal distances in virtual space. While ties were observed at discrete weekly sessions, SAOMs conceptualize tie formation as a continuous process in which an observed network state reflects the accumulated result of many unobserved micro-level decisions occurring between observations, providing justification for using the mode of proximity across each 30-minute session as an indicator of the relational state aggregated over the preceding week [68]. While all interactions took place in one large open space (“Engineering Workshop”), the use of 3D voice in the ENGAGE platform allowed for splitting off into smaller groups without audio overlap. As the volume of a person’s voice reduces as the distance to them increases (no volume beyond 15 meter by default), participants had to physically move closer to their small group members to hear and communicate with each other. As seen in S1 Fig, the distribution of the interpersonal distances ($distance_{ijt}$) exhibited a bi-modal distribution with modes at approximately 1.5 and 8 meters and a clear trough at approximately 4 meters (*M* = 7.15, *SD* = 3.86; 1st quartile = 3.19, 2nd quartile [*Mdn*] = 7.80, 3rd quartile = 10.14). Drawing from some definitions used in proxemic theory [69,70] about individuals’ perceptions of intimate space (0–45 cm), personal space (45–120 cm), social space (129–365 cm), and public space (365–762 cm) and the observed empirical distribution we defined the presence/absence of social ties between participants using a 400 cm (4 meters) threshold. Specifically, each tie variable $x_{ij}$ was coded as present = 1 when $distance_{ijt}<4$ and absent = 0 when $distance_{ijt}\geq 4$ or missing. Of the 66,761 distance measurements obtained over the five weeks, 17,108 (25.6%) were less than 4 meters. The resulting 40 binary networks (8 groups × 5 weeks) indicate which pairs of participants engaged in meaningful social interactions in the CVE classroom and how those social ties changed over time. The same pattern of results was obtained in follow-up robustness checks using a 365 cm threshold of social space (see S2 Table).

#### Logic of tie changes.

When there is an opportunity for change actors’ decisions are guided by the objective function. However, there are different ways that actor’s decisions are combined in creating, dissolving, or maintaining ties. For example, in a dictatorial logic one actor makes a decision and unilaterally imposes the change in tie. In other logics, one actor takes the initiative and proposes a change to the second actor who may agree or not, or both meet and reconsider the tie together. For non-directed networks, it is commonly assumed that changes in ties involve the mutual consent of both actors [59,61].

In the “unilateral initiative and reciprocal confirmation” logic, actor *i* takes the initiative, choosing the best possible $x_{ij}$ to change. If the proposed action is to dissolve the tie, the change is carried out. If the proposed action is to form a new tie, the new tie is proposed to *j*. If *j* agrees, then the change is carried out, otherwise nothing happens. In our analysis, we invoked this logic, where tie changes are initiated by one actor and tie formations require consent (“modelType = 3”, see [57]).

### Stage 2: Model definition

The SAOM framework allows researchers to specify a range of factors, structural and individual- or dyad-level covariate effects as shown by Fig 2, in the rate function (modeling occurrences of change) and objective function (modeling mechanisms of change). As in prior work, we assumed that the rate function only depends on period and that all actors in a given group network have an equal probability of change in Eq. (1). In defining the objective function in Eq. (2), we introduced three models to investigate how different interaction measures influence tie formation and change in VR: Model 1 (Structural and Actor effects), Model 2 (Model 1 + Actor-partner dissimilarity effects), Model 3 (Model 2 + Dyadic similarity effects).

Model 1 presents a basic model specification that includes two structural effects and six individual-level actor covariate effects in the objective function. Derived for each weekly group network, two structural effects represent essential network characteristics that are related to bridging and boding social capital. As a measure of baseline tendency, out-degree ($\beta_{\text{OD}}$) shows how extensively an individual is connected to others in the group, or how dense their network is. As a measure of social capital, GWESP ($\beta_{\text{GWESP}}$) captures how likely they are to form transitive ties with friends of their friends. This effect investigates how bridging (negative GWESP) and bonding (positive GWESP) structures would manifest in virtual classroom settings.

Also, individual difference measures drawn from weekly surveys were included as baseline covariate effects. In line with the previous work [6], we hypothesized that in VR classrooms, participants’ prior experience with VR ($\beta_{\text{priorVR}}$), prior acquaintance with group members ($\beta_{\text{priorAcq}}$), and group identification ($\beta_{\text{groupIdn}}$) measured in the pre-test questionnaire, as well as time metric ($\beta_{\text{time}}$), may influence their networking behavior in each weekly group network. These actor effects investigate whether and how individuals’ prior VR- or group-related experiences influence their actual participation in VR group activity, and whether these group dynamics evolve over time.

We also included two possible extensions that allow for a more nuanced examination of actor-partner interaction and dyadic interaction, respectively. Model 2 specifies how actor-partner imbalance in individual difference measures may influence VR interaction behavior. We hypothesized that increases in actor-partner imbalance (absolute sum of differences) across the three time-invariant individual difference measures, that is, prior VR experience ($\beta_{\text{priorVR.Imb}}$), prior acquaintance with group members ($\beta_{\text{priorAcq.Imb}}$), and group identification ($\beta_{\text{groupIdn.Imb}}$), would decrease the likelihood of tie formation. Additionally, considering that participants may have drawn demographic information from voice cues and that they were in the same class for 10 weeks, we include the same gender ($\beta_{\text{gender}}$) and same ethnicity ($\beta_{\text{ethnicity}}$) effects to test homophily based on demographic background. Model 3 incorporates two time-invariant dyadic relationship measures obtained from the post-test questionnaire. Specifically, we assessed whether prior familiarity ($\beta_{\text{familiarity}}$) and friendship ($\beta_{\text{friendship}}$) with group members increase the likelihood of tie formation. These actor-partner and dyadic effects investigate how bridging (positive dissimilarity, negative similarity effects) and bonding (negative dissimilarity, positive similarity effects) structures would manifest in VR interaction.

### Stage 3: Model estimation and selection

All three models were estimated using the R “RSiena” package (version 1.4.7) [57]. The eight groups were analyzed simultaneously using the multigroup option, which assumes the groups are independent replicates with their own group- and period-specific rate parameters (see S1 Table), but a common objective function. The multigroup model was specified using the initiative with reciprocal confirmation logic of tie changes (“modelType = 3”; see modelType = 2–6 for non-directed networks, [57]). Multigroup SAOMs were then fitted using the conditional MoM method, with 3,000 iterations in the third phase, a threshold of 0.25 for the overall maximum convergence ratio, and a threshold of 0.1 for the absolute value of the individual *t*-statistics. Model comparisons (e.g., Model 2 vs. Model 1, Model 3 vs. Model 2) were done using Wald-type chi-square tests. As a robustness check, in S1 Table we calculated Jaccard similarity measures, where values > .3 are preferred; values < .1 should be treated cautiously [11,57,68].

## Results

This study used SAOMs to explore fundamental processes of social interaction in CVEs, where individuals were deliberately transported into VR to engage in group learning activities and establish social relationships with peers. The results are presented in two sections. We begin with a descriptive analysis of VR group networks, then examine patterns of bridging and bonding structures found in SAOM estimation results. To provide a comprehensive illustration of the SAOM framework, we also discuss findings regarding the structure and evolution of virtual community.

### Descriptions of VR group networks

**Fig 3:**
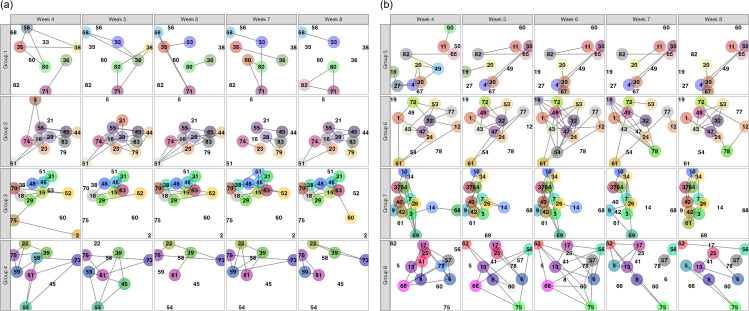
Observed weekly group networks based on interpersonal distances in VR. Each node denotes a participant (i.e., actor) in VR classroom, and each edge indicates presence of close-distance interaction (i.e., tie) between participants. Two actors, A and B, who are in the same group have a social tie in a given week if both A and B are present in VR classroom that week and their pairwise in-VR interpersonal distance < 4, where “4” is the median value of pairwise distances in the data. If either A or B was absent from class, or if the distance between the pair {A, B} was greater than or equal to 4, A and B are considered as not connected to each other. The symmetric (non-directional) adjacency matrix indicating the ties among members of each group for each week is then used in the analysis. Node placement in the figure is based on the Fruchterman-Reingold force-directed layout algorithm across weeks. Actor nodes are colored when that actor has at least one tie each week. (a) Groups 1-4. (b) Groups 5-8.

Fig 3 illustrates the evolution of social ties across five weekly VR sessions (in columns) within the eight discussion groups (in rows). Two consistent patterns emerged across these longitudinal group networks. First, we observed a general trend of network decay (rather than growth). Both the number of active participants (actors) and the number of social ties decreased over time, implying that participants were less likely to interact with peers and more likely to disengage with the classroom setting over time. For example, in Group 1, the number of distinct ties decreased from 6 (among 6 actors) in Week 4 to 4 (among 5 actors) in Week 8. Similarly, in Group 2, the number of distinct ties decreased from 11 (among 10 actors) in Week 4 to 8 (among 9 actors) in Week 8. Across groups, average network density decreased from .17 (*SD* = 0.07) in Week 4 to .11 (*SD* = 0.03) in Week 8. S3 Table illustrates how each discussion group’s network densities decrease and change week-to-week across the five weeks.

Second, we observed that while the overall size and density of group networks declined over time, the cohesiveness within smaller clusters tended to persist. Individuals stayed connected to their subgroups. In Group 5, for example, eight (out of 10) participants initially formed a core-periphery structure that consisted of a dense core (Actors #49, #20, #30, #4, #27) and a sparse periphery (Actors #82, #65, #19) in Week 4, but this network was gradually separated into three small subgroups by Week 8, one of which (Actors #30, #50, #4) pretty much remained intact across the four remaining weeks. Similarly, in Group 6, the one loosely connected network that existed in Week 4 had split into three fully connected subgroups by Week 8 (Actors #72, #12, #78; Actors #1, #47, #24; Actors #49, #43, #47, #32). These patterns of differential network decay – ties dissolve globally but persist locally – motivated our use of SAOMs to examine how bridging and bonding mechanisms contributed to tie formation, maintenance, and dissolution in VR classrooms.

### SAOM estimation results

Following the procedures outlined above, we fitted three multigroup SAOMs (Models 1–3) that incorporated incrementally larger sets of structural and covariate effects. As seen in the bottom rows of Table 2, all three models converged; overall maximum convergence ratios were below .25 and absolute values of the individual *t*-statistics were below .1. Based on the model comparison using Wald-type chi-square tests, we selected Model 3 as the best fitting model (Model 1 → 2, $\chi^{2}(5)=15.26$, *p* < .001; Model 2 → 3, $\chi^{2}(2)=33.33$, *p* < .001) and thus focused interpretation on these results.

**Table 2:** Results from SAOMs examining how structural, individual-level covariate, and dyad-level covariate effects contribute to network change.

	Model 1	Model 2	Model 3
Parameter	Actor effects	Actor-partner effects	Dyadic effects
**Structural effects**			
Out-degree	−0.835*** (0.123)	−0.757*** (0.170)	−0.638*** (0.174)
GWESP	0.898*** (0.113)	0.917*** (0.110)	0.863*** (0.115)
**Individual-level covariate effects**			
Time	−0.036 (0.034)	−0.036 (0.033)	−0.057 (0.035)
Prior VR use	−0.098 (0.117)	−0.099 (0.136)	−0.155 (0.141)
Prior acquaintance	0.007 (0.124)	0.081 (0.130)	−0.168 (0.149)
Group identification	−0.022 (0.060)	−0.075 (0.058)	−0.094 (0.060)
Prior VR use imbalance^a^		0.183 (0.117)	0.155 (0.111)
Prior acquaintance imbalance^a^		−0.185 (0.124)	−0.051 (0.128)
Group identification imbalance^a^		−0.174** (0.063)	−0.174** (0.062)
Same gender		0.139 (0.097)	−0.015 (0.105)
Same ethnicity		−0.086 (0.122)	−0.154 (0.124)
**Dyad-level covariate effects**			
Familiarity			0.368*** (0.068)
Friendship			−0.055 (0.063)
**Goodness-of-fit statistics**			
Chi-square statistic (*df*) ^b^		15.261*** (5)	33.334*** (2)
Overall maximum convergence ratio	.097	.120	.123
Absolute maximum of *t*-statistics	.068	.054	.049

GWESP = geometrically weighted edgewise shared partners. Multigroup models fit by the conditional Method of Moments estimation with 3,000 iterations in the third phase, using the “RSiena” package (version 1.4.7). Model convergence is obtained when the overall maximum convergence ratio < .20 and the absolute value of individual *t*-s*t*atistics < .10. Parameter estimates with standard errors in parentheses. Statistically significant parameters indicated in **bold** font. *p < .05. **p < .01. ***p < .001. ^a^Imbalance is measured by the absolute sum of differences between a focal actor and its partners. ^b^Tests null hypothesis that newly added parameters are zero.

#### Rate function.

The rate parameters are typically considered as nuisance parameters [57]. In our case, they also do not inform any of the hypotheses about social capital theory. For completeness of reporting, rate parameter estimates from Model 3 are shown in S1 Table, where we see that the separately estimated rate parameters exhibit an overall decreasing trend. This trend in part reflects how the networks decay over time.

#### Objective function.

The estimated coefficients in Table 2 correspond to the effects on the probability of tie formation and change, capturing the rules of change defined by each model’s objective function. These parameters are assumed to be equivalent across all eight groups. The sienaTimeTest() results showed that the out-degree effect is not significantly different across 32 weekly group networks and across eight groups ($\chi^{2}(31)=12.58$, *p* = .999 in Model 1; $\chi^{2}(31)=14.05$, *p* = .996 in Model 2; $\chi^{2}(31)=32.67$, *p* = .384 in Model 3).

**Fig 4:**
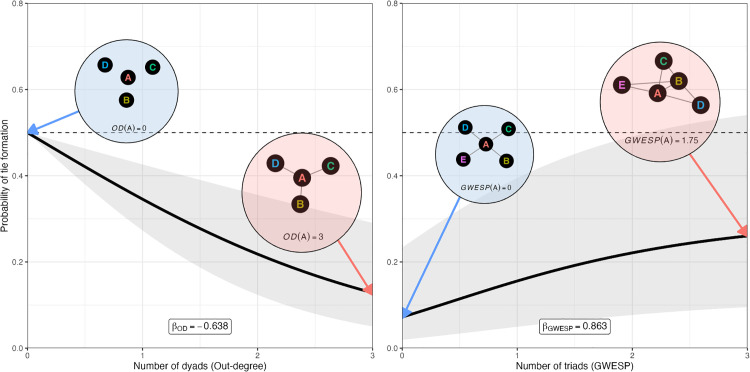
Structural effects on tie formation and change. Out-degree effects on the left and GWESP effects on the right, based on the best fitting Model 3. Each of four networks showcases out-degree or GWESP values for actor A when OD(A) = 0, OD(A) = 3, GWESP(A) = 0, GWESP(A) = 1.75 (from left to right), respectively. On the right panel, when the number of actor A’s triads is 0 (i.e., GWESP(A) = 0), the probability of tie formation is less than 0.5 because actor A already has four ties (i.e., OD(A) = 4). When the number of actor A’s triads is 3, GWESP(A) = 1.75 < 3 because GWESP measures triad census with an additional decaying parameter α = ln(2), meaning that adding partner is weighted downwards as the number of existing triads increases.

**Structural effects:** The negative out-degree effect ($\beta_{\text{OD}}=-0.64$) indicates that individuals tended to interact with smaller, often selective, groups: each additional tie reduces the likelihood of forming a new tie by about half (i.e., exp(−0.64)=0.53). In contrast, the positive GWESP effect ($\beta_{\text{GWESP}}=0.86$) suggests a more than 2 times stronger tendency to build on their smaller subgroups (i.e., exp(0.86)=2.36). If both actors have high GWESP, it can push even more strongly toward the formation of a new tie, reinforcing bonding ties within subgroups. Fig 4 displays structural effects on tie formation and change when all other covariates are controlled in Model 3. For example, on the right panel of Fig 4, when there is no transitive relationship among any of actor A’s four ties with B-F, the probability of finding a new partner is below 0.1 due to the negative out-degree effect: 1/(1+exp(−(−0.64×4)))=0.07. However, the presence of a shared third connection (i.e., triad) significantly increases the likelihood of tie formation and change, with more triads amplifying this tendency. For example, for the tie between actors A and B where they share three common friends (C, D, E), it is about 4 times more likely that they will connect to the friend of these three friends rather than a random person: 1/(1+exp(−(−0.64×4+0.86×1.75))))=0.28.

These findings align with our descriptive analysis, suggesting that even as network size and density decrease over time, potentially due to classroom settings, participants tended to connect with their smaller, cohesive subgroups. In other words, participants were more likely to maintain bonding ties with their “cliquey” peers, rather than to form new connections – such as engaging with “popular” others – that would support bridging. From the classroom design perspective, these results underscore the importance of triadic structures in encouraging student participation in CVEs.

**Fig 5:**
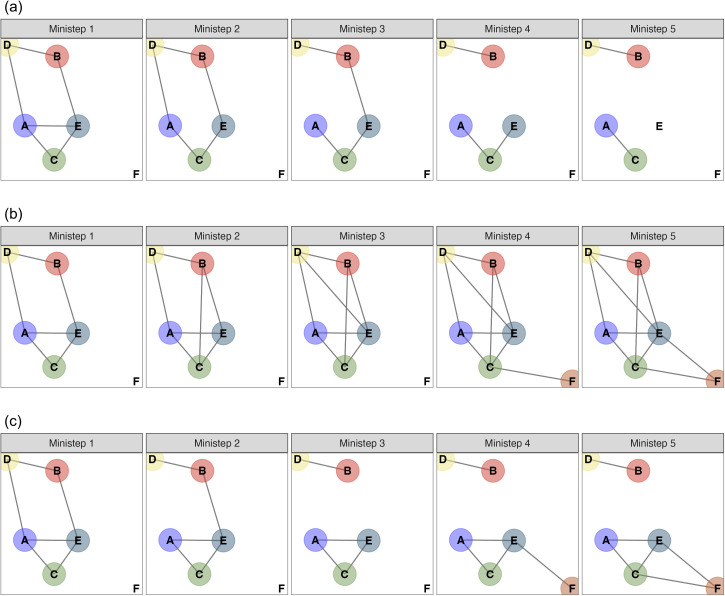
Simulated network change under different configurations of structural effects. Examples of simulated networks based on the best fitting Model 3, where $\beta_{\text{OD}}=-0.64$ and $\beta_{\text{GWESP}}=0.86$. Based on an example group network at Ministep 1 with six actors A-F, predicted tie changes over four ministeps are displayed using three scenarios (for simulated week-to-week network change, see S2 Fig). (a) If there is negative out-degree effect and no GWESP effect (i.e., $\beta_{\text{OD}}=-0.64$ and $\beta_{\text{GWESP}}=0$), the density and size of the network will decrease over time. This scenario shows that negative out-degree effect discourages people from bridging between communities and creating new contacts. (b) If there is positive GWESP effect and no out-degree effect (i.e., $\beta_{\text{OD}}=0$ and $\beta_{\text{GWESP}}=0.86$), then the network will become dense, specifically due to additional connections among friends of friends. This scenario shows that positive GWESP effect encourages people to derive relations from and build stronger relationships within the same group. (c) Finally, Model 3 (i.e., $\beta_{\text{OD}}=-0.64$ and $\beta_{\text{GWESP}}=0.86$) predicts that the loosely connected one group network will be divided into small subgroups with close bonds. The other covariate effects are fixed to be zero (i.e., no effect due to covariate differences).

To better understand the role of structural effects in network dynamics, Fig 5 illustrates three hypothetical scenarios: The top panel simulates evolution of a network with a negative out-degree effect and no GWESP effect ($\beta_{\text{OD}}=-0.64$, $\beta_{\text{GWESP}}=0$); the middle panel simulates evolution of a network with a positive GWESP effect and no out-degree effect ($\beta_{\text{OD}}=0$, $\beta_{\text{GWESP}}=0.86$); and the bottom panel simulates evolution of a network with a negative out-degree effect and positive GWESP effect, as observed in Model 3 ($\beta_{\text{OD}}=-0.64$, $\beta_{\text{GWESP}}=0.86$). Across the five consecutive ministeps, the first scenario shows that, without any bonding ties, inhibitory bridging processes lead to sparse, loosely connected networks. In complement, the second scenario illustrates how, without any bridging ties, bonding processes drive the formation of dense, close-knit networks. As a combination of these two processes, the third scenario captures the actual pattern in observed data: an initially bigger, loosely connected network evolves into smaller, close-knit subgroup networks over time. These scenarios suggest that a more ideal classroom environment may emerge by sustaining these subgroup networks in ways that allow for both bridging and bonding processes.

**Individual-level covariate effects:** None of the four actor effects were significant. Although these directions suggested an overall negative pattern – the odds of creating a new tie decreased over time ($\beta_{\text{time}}=-0.057$, *p* = .098), with lower probabilities among participants who have prior VR experience ($\beta_{\text{priorVR}}=-0.155$, *p* = .272), who have prior acquaintances within their assigned VR discussion groups ($\beta_{\text{priorAcq}}=-0.168$, *p* = .261), and who have above average identification scores to the discussion groups they belong to ($\beta_{\text{groupIdn}}=-0.094$, *p* = .119) – none reached statistical significance.

Among the five actor-partner effects, only group identification imbalance showed a significant effect: greater imbalance in group identification was related to a reduced likelihood of tie formation ($\beta_{\text{groupIdn.Imb}}=-0.174$, *p* = .005). This finding suggests that participants were more likely to connect with others who shared similar levels of group affiliation, in support of bonding social capital. This may be particularly relevant given that our sample included participants who tended to have higher group identification and to enroll in the same group (e.g., student athletes in the same team). No such effects were found for prior VR experience ($\beta_{\text{priorVR.Imb}}=0.155$, *p* = .162), prior acquaintance ($\beta_{\text{priorAcq.Imb}}=-0.051$, *p* = .693), same gender ($\beta_{\text{gender}}=-0.015$, *p* = .885), and same ethnicity ($\beta_{\text{ethnicity}}=-0.154$, *p* = .214).

**Dyad-level covariate effects:** Among the two dyadic effects, familiarity was positively associated with tie formation ($\beta_{\text{familiarity}}=0.368$, *p* < .001), whereas friendship was not ($\beta_{\text{friendship}}=-0.055$, *p* = .385). This finding that participants were more prone to interact with familiar others is also indicative of a bonding process.

Taken together, effects associated with bonding mechanisms – GWESP and similarity-based covariate (i.e., familiarity) effects – were positive, while effects associated with bridging mechanisms – dissimilarity-based covariate (i.e., group identification imbalance) effects – were negative. In conclusion, bonding capital, rather than bridging capital, manifested in these data obtained in a study of groups interacting in a CVE across multiple weeks during a university-based VR class.

## Discussion

In this paper, we developed a methodological framework that combines longitudinal network methods with VR tracking data. Specifically, the SAOM approach has the potential to uncover social dynamics of “virtual community” in CVEs, through the lens of bridging and bonding social capital. We illustrated the method through analysis of a longitudinal dataset on interpersonal distances obtained over five weeks during a university course about VR. First, we conducted an exploratory analysis of distance-based social networks across groups and weeks, noting a general trend for differential network decay over time. Second, we used the SAOM to examine a variety of possible effects that indicate how social capital manifests in the CVE classrooms being studied. We found that bonding, rather than bridging, structures manifested in VR classrooms using the structural effects, as indicated by the positive GWESP effect. Evidence of bonding social capital in this virtual classroom settings also emerged in covariate effects, particularly through the negative actor-partner effect of group identification imbalance and the positive dyadic familiarity effect.

In the sections that follow we examine the social structure of virtual community. We then probe how evidence of social mechanisms can be leveraged to create inclusive and accessible CVEs. Finally, we discuss contributions of developing a SAOM framework in applications and studies of VR as well as other communication technology.

### The social structure of virtual community

Our empirical analysis has three main findings. First, a general trend of network decay illustrates that participants were less likely to interact with peers and more likely to disengage with the classroom setting over time. Although disengagement over time has been documented in online learning environments more broadly, the mechanisms underlying our finding likely differ. For example, student silence typically concerns the verbal exchange with lecturers [71], whereas our finding concerns the nonverbal distancing behavior among peers. Several explanations may account for this pattern. One possibility is that as the VR medium became less novel over the course of the study, participants may have felt less motivated to explore and interact within the CVE. Also, because sparse conversation was not penalized in the grades, they were less incentivized to actively engage with peers. It is also worth noting that the specific topics and activities varied from week to week. Distinguishing among these possibilities remains an important direction for future work. Second, as individuals stayed connected to their subgroups, the cohesiveness within smaller clusters tended to persist (positive GWESP effect). This finding is consistent with the tendency of social VR platforms to host numerous, relatively small, communities with strong internal cohesion and limited external connections [72]. For example, in a similar study of VR workshop settings, participants occupied a relatively small area, even when there was plenty of room to spread out [73]. Third, individuals were more likely to connect with others who shared similar levels of group affiliation (negative group identification imbalance effect) or were already familiar (positive dyadic familiarity effect). This finding aligns with the literature on homophily in social VR contexts, where friendships tended to rely heavily on familiarity, common interests, comfort in spending time together, and feeling close to others with such characteristics [74]. For example, groups of friends reported higher copresence, showed more positive sentiment, and engaged more frequently in dyadic interactions than the groups of strangers [75]. However, participants did not show a significantly stronger tendency to connect with others who share the same gender or ethnicity. These results support the previous finding in the same VR dataset that demographic characteristics did not predict student outcomes [6]. While this pattern should be interpreted with caution given the exploratory nature of the analysis, it is consistent with the possibility that VR settings – wherein users can choose avatars to visually represent themselves – may reduce social boundaries across demographic groups [76–78]. The question of whether and how VR can attenuate ethnic or gender biases in teaching and learning settings should propel additional research in this area.

While our dataset does not include offline interactions data, prior studies found that students tended to build stronger relationships with “friends of friends” in in-person classrooms [33–35]. Similarly, our empirical results suggest that these bonding dynamics also manifested in VR classrooms [42,43,79]. This suggests that the techniques used to foster bonding in traditional or online educational settings can be adapted into VR settings, although empirical validation is needed. For example, inviting existing friends or assigning groups based on shared interests or past interactions might help users onboard in VR settings [74]. We note that our findings apply to interpersonal distancing behaviors in VR classrooms specifially, and that direct comparison to in-person classroom dynamics needs more exploration that is beyond the scope of this study.

Unlike the existing literature on bridging structures of online community [37–39], we found little evidence that actors were inclined to bridge with interaction partners who can expand their social reach. As outlined by Yuan and Gray [32], bonding social capital contributes to strengthening relationships and increasing group cohesiveness, but it also divides a network into smaller cliques. This opens two possibilities. First, participants may have been unfamiliar with the VR setting – about 50% of the participants had never experienced VR – which may have limited these participants ability to engage bridging social capital. While bonding processes with existing friends can draw on shared context and rapport, bridging processes with new friends often require higher levels of presence and embodiment to overcome initial uncertainty – making it more challenging for new users to make use of bridging social capital. Second, interaction dynamics in VR may fundamentally differ from those in both physical and online environments. Maloney et al. [80] suggests, while nonverbal behaviors and social norms are quite similar in the virtual world (vs. physical world), they are still different in many ways. For instance, walking up to a stranger to bump their fist in the real world might be different from “gliding” to a stranger to extend their “hand controller” in the virtual world. Because VR technology affords different modes of interaction, even similar gestures like greeting can carry different meanings in the virtual world. As such, the same interpersonal distancing behavior may signal something different in VR than it would in the physical world.

**Fig 6:**
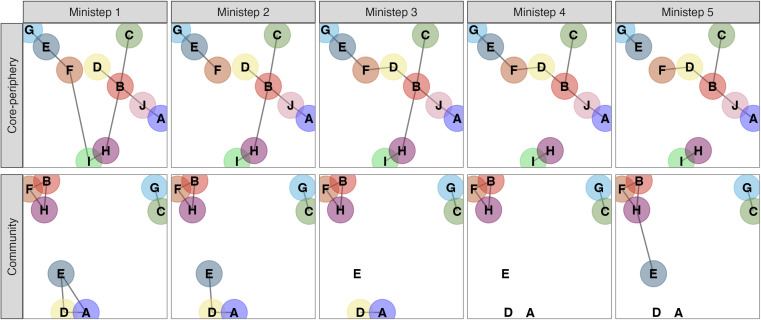
Simulated network change under core-periphery and community structures. Examples of simulated networks under core-periphery- and community structures. Based on an example group network at Ministep 1, predicted tie changes over four consecutive ministeps are displayed for each network structure (for simulated week-to-week network change, see S2 Fig). Node placement in the figure is based on the Fruchterman-Reingold force-directed layout algorithm across weeks. Actor nodes are colored when that actor has at least one tie each week. Top panel: Under the core-periphery structure, the dense core (i.e., node B) is more likely to remain well connected with adjacent nodes, while peripheral nodes are only sparsely and loosely connected. Bottom panel: Under the community structure, cohesive subgroups are less likely to be united into bigger networks, or even expand their subgroup network, as tie formation primarily occurs within, rather than between, groups.

Although we do not have a definitive answer between the two possibilities, our analysis consistently suggests that bonding, rather than bridging mechanisms were more prominent in VR networks. Using the parameters from Model 3, Fig 6 shows how two prototypical network structures – a core-periphery structure in the top row and a community structure in the bottom row [50] – tend to fragment into smaller subgroups over four ministeps. In the core-periphery structure (top row), central nodes are more likely to terminate their links with peripheral nodes (e.g., Week 4 → 5), separating core and peripheral subgroups. In the community structure (bottom row), given that the three communities were already fully connected in Week 4, it follows that the network grows only sparser, without additional tie formation in the following weeks. We note that these are theoretical illustrations of the model’s equilibrium properties using only four ministeps. The number of ministeps may be different in reality, so these simulations are illustrative and should not be interpreted as specific predictions of how an actual group with those starting structures might evolve.

In essence, this model-based simulation highlights the role of bridging ties in network dynamics, suggesting that in VR classrooms, it may be particularly important to foster interactions with a wide range of group members outside their existing subgroup or familiar others. One possible approach is implicitly shaping group activities so that students are naturally brought into bridging with unfamiliar others rather than only familiar ones. For example, on Week 6, participants engaged in small-group activity in which participants chose a unique feature of VR and brainstormed how to communicate climate change based on this feature. To facilitate bridging processes, such an activity could be structured so that participants spend the first half with two partners of their own choice and then rotate to a new set of partners, also of their own choice, which would naturally expand their reach beyond their familiar subgroup. Rather than explicitly pairing students with specific unfamiliar others, this implicit shaping of group activities and learning environments would encourage students to engage with bridging processes without overriding their individual choices and preferences. Although empirically testing such interventions is beyond the scope of the current study, future studies could examine whether and how interventions focused on fostering bridging processes can facilitate more inclusive network dynamics in VR.

Marginalization in CVEs, also referred to as “virtual ostracism” [81], is a growing concern for design of more open and inclusive communities. While our analyses found no homophily based on actual gender or ethnicity in the VR setting, marginalization may implicitly and explicitly shape avatar-mediated interaction in social VR [82,83] and thus remains a broader structural issue. As illustrated in Fig 6, our analysis suggests that bonding social capital in the absence of bridging may be a fundamental source of this marginalization. These findings also have important implications for facilitating long-term VR group interactions. For example, when familiarity mediates intergroup contact [84], interventions leveraging existing similarity and shared preferences may successfully facilitate emergence of positive intergroup attitudes [85]. Extending from cross-sectional intervention studies [85,86], our study illustrates how longitudinal data and methods can be used to track how the network dynamics evolve over time.

### Network approach to VR tracking data

In this paper, we demonstrated how SAOMs can be used to examine social dynamics in VR classrooms. The SAOM framework provides a practical solution for bringing network perspectives to VR. Moreover, this framework can be extended to incorporate a wide variety of structural, individual- and dyad-level covariate effects to capture bridging and bonding social capital. As such, this study proposes and begins developing an empirical research agenda – the longitudinal network approach facilitates an exploration of webs of personal relationships, or “virtual community” that has been an implicit premise in the field since its introduction [5], but yet to be directly tested or implemented. By adding dyads, groups, communities, or societies as another layer of analysis, above the existing layer of individuals, that highlights the importance of social dynamic. Advances in VR technology that make virtual environments feel more like “reality” prompted study of whether individuals behave similarly in VR and in the physical world [1–4]. However, many existing research are based on individual or dyadic interactions in VR, leaving a substantial gap between the empirical analysis of the longitudinal data obtained in CVEs – including triadic and group-level features (e.g., gaze, motion synchrony) – and Rheingold’s concept of virtual community as webs of personal relationships. Our longitudinal analysis applies SAOM methods to VR motion data to begin addressing this gap, examining whether patterns of bridging and bonding structures in a VR classroom setting are similar to those found in the previous literature in in-person classrooms.

The application of the SAOM network method is not limited to only VR motion data. Taking advantage of the latest tracking technology underlying CVEs, rich and real-time tracking data collected in the virtual world can be easily incorporated into the SAOM analysis. To illustrate, voice recordings can be used to quantify lexical similarity between individuals; eye gaze cues can be considered as a measure of selective and multi-directional attention. Taken together, researchers can make use of verbal and nonverbal communication behaviors in multiple modalities and potentially in combination of each other, to represent connections between people. As immersive VR technology continues to develop, we argue that network-based approach is an essential and promising method for building more accessible and inclusive learning environments – that can also support meaningful social interactions in computer-mediated contexts – and developing interpersonal relationships in VR.

## Limitations and outlook

The empirical example here had the goal to developing and illustrating how SAOM methods can be used to study social dynamics in VR, with a particular emphasis on interpersonal distancing [27,28]. However, our operationalization has several limitations surrounding interpretation of interpersonal distance in VR. We discuss three limitations. First, we employed a threshold approach to derive binary networks of distance < 4-meter (or not). However, each week of the study, participants engaged in a variety of learning tasks across different topics and various virtual environments [6]. The specific distance threshold therefore may not capture actual relationship webs across all weeks, activities, and environments. While we obtained the same pattern of results in follow-up robustness checks using a 3.65-meter threshold (as in proxemic theory, see S2 Table), dichotomization in the design of binary networks may require more theoretical and empirical testing [87]. Second, the distance measure was defined as the mode of distances across all 1/3-meter spatial bins and 1/30-second temporal epochs (30 Hz) of every 30-minute VR session. Although the mode was particularly used to represent the distance at which participants spent the most during the 30-minute session, alternative metrics such as mean or median, in combination with varying sizes of spatial bins and temporal epochs, can be employed for further robustness checks. Third, unlike many applications in the SAOM literature, our networks are non-directed, which is inherent in the use of non-directional distance. As the data allow for detailed positional and rotational data, future works can incorporate the asymmetry of multi-modal and multi-directional interactions among dyads, such as head directions or eye gazes.

Combined with the increasing availability of VR motion and tracking data, our SAOM approach can be extended for conceptualizing and quantifying how ties change moment-by-moment and week-to-week. Importantly, the choice of time interval (i.e., weekly VR sessions) – ranging from 1/30-second to 30 minutes – involves both conceptual and practical considerations in modeling network change. For example, by calculating ties every 10 minutes within each VR session, the resulting tie changes can be more transient and dynamic than those typically used in the SAOM literature (e.g., classroom seating charts or online friendship networks). Conceptually, the time unit should be relevant to the construct underlying the tie variable, that is, social relationship underlying VR interaction. In the analysis, we calculated the mode at the 30-minute session level because each weekly session was designed around one activity, but researchers should think carefully about whether their chosen time unit accurately reflects the nature of social ties. Practically, the time unit should align with the SAOM model assumptions. For example, a tie variable defined at the 1/30-second epoch level is subject to physical dependence, which can violate single-tie-change assumption. In such cases, alternative spatial spatial or interaction-based models can be pursued to study VR interaction at the epoch level [88,89]. Additionally, tie change processes are assumed to be time-homogeneous in the SAOM approach [11], which can require additional time-varying covariates to account for participants’ changing perceptions or attitudes underlying their VR interactions.

Based on our operationalization, we propose elaborating the modeling in several ways. First, for model selection, we incrementally added two set of covariates to examine various interaction measures in Models 2 and 3, then tested whether each addition significantly improved model fit. While our approach follows standard model comparison procedures, alternative methods exist that emphasize theoretical relevance [11] or address methodological issues (e.g., MDMC test statistics, time heterogeneity) [58]. Second, multigroup SAOM analysis relies on the strong assumption that all structural and covariate effects (although not rate parameters) are equal across groups and weeks. Also, in our motivating example, we make use of the 4-meter threshold for defining a social tie, which is the median distance across all eight groups and five weeks. Because this is a set of strong unrealistic assumptions that completely ignores group differences (e.g., distance thresholds might differ by group and also change over time), future research may consider ongoing developments in the SAOM approach. Specifically, recent modeling approaches to multilevel network data, including meta-analysis or multilevel analysis including Bayesian and random coefficient approaches [59,90,91], can be useful. For example, the sienaBayes() function in the multiSiena package is designed to handle small groups under relaxed homogeneity assumptions. Third, while SAOMs are not built for weighted networks (only binary networks), it is possible to specify structural zeros (or ones) that indicate when actors leave or join the network [57]. In this way, we can represent the network’s changing composition in a classroom setting, for example, students who change their group or drop the course in the middle of five sessions. Fourth, the framework can be expanded to include more time-invariant/varying covariates and their interaction terms and to model the co-evolution of networks and behavior [55]. In this paper, we used individual difference measures and demographic background in the pre-test questionnaire data as predictors of tie formation and change. Considering that participants were allowed to personalize their avatars, future studies can examine if and how homophily based on avatar personalizations as well as additional demographic variables (e.g., age, race) manifests in VR contexts [42–45]. Furthermore, by interacting these covariates with structural effects, we can test further social capital hypotheses, for example, whether prior VR use moderates the GWESP effect. Supplementing these time-invariant covariates, time-varying covariates can be added and modeled as co-evolutionary dependent variables, allowing for studying temporal patterns of covariate effects. Fifth, in illustration of dyad-level covariate effects, we included familiarity and friendship variables that were both obtained at the post-test questionnaire. These variables were used because they were the two available dyadic relationship measures where participants rated each member in their group as part of the course evaluation. However, these measures are treated here as time-invariant predictors of network change and thus should be interpreted as associated factors and not as causal factors. Finally, our model specifications included evaluation effects only, modeling the overall process of tie formation and change. Researchers specifically interested in tie persistence should consider splitting parameters into creation and endowment effects [57].

## Conclusion

Our analysis highlights the exciting opportunities VR motion data provide for fine-grained study of small group dynamics. In this paper, we analyzed longitudinal data from a university course taught in VR to demonstrate how SAOMs can be used to track and investigate network dynamics in CVEs. Our approach addresses gaps in previous research through the investigation of network structural effects and individual- and dyad-level covariate effects that allows for measuring bridging and bonding processes associated with social processes in VR. The SAOM method is a sociological theory-driven and scalable framework that can be applied to many different types of newly available VR tracking data. Pioneering integration of sociological network theories with the traces of cutting-edge VR technologies opens new possibilities for innovative study of group behavior and community formation. We hope that this first application and demonstration of SAOMs to VR motion data will drive more discussion and empirical analysis of social networks in the computer-mediated environments.

## Supporting information

S1 Text Rate function and objective function in SAOMs. (DOCX)

S1 Fig Histogram of the interpersonal distances observed across multiple weeks in the CVE. Blue dashed line at 4-meter. The distance measure exhibited a bi-modal distribution having two modes at approximately 1.5 and 8 meters and a trough at approximately 4 meters (*M* = 7.15, *SD* = 3.86; 1st quartile = 3.19, 2nd quartile [*Mdn*] = 7.80, 3rd quartile = 10.14). Of the 66,761 measurements across all group members over the five weeks of study (Weeks 4–8), 17,108 (25.6%) measurements were less than 4 meters.
(TIFF)

S2 Fig Model-based simulations of week-to-week network change. An example run of 3,000 simulated networks (i.e., 3,000 iterations in the third phase) based on observed data. Week 4 is the baseline week and not included in prediction. Node placement in the figure is based on the Fruchterman-Reingold force-directed layout algorithm across weeks. Actor nodes are colored when that actor has at least one tie each week. (a) Groups 1–4. (b) Groups 5–8.
(TIFF)

S1 Table Rate parameter estimates from SAOMs. (DOCX)

S2 Table Results from SAOMs examining how structural, individual-level covariate, and dyad-level covariate effects contribute to network change: Using 3.65-meter threshold for tie presence. (DOCX)

S3 Table Descriptive statistics of observed weekly group networks. (DOCX)

S1 Appendix Supplemental R Scripts based on RSiena. (HTML)
