# Supplementary material for: Evolution of networks in virtual reality: A scalable framework for studying social dynamics of small groups
Source: PLoS One. 2026 Aug 11;21(8):e0355470. doi: 10.1371/journal.pone.0355470 (PMC13460626; doi:10.1371/journal.pone.0355470)
Supplement: Supplementary file 1 [file pone.0355470.s001.docx]

**S1 Text**

*Rate function and objective function in SAOMs*

**Rate function*.*** The *rate function* describes the expected number of opportunities that an actor gets to change (form or dissolve) one of their outgoing ties. Formally, actor *i*’s waiting times until the next ministep – the opportunity for change – in a given period between two actually observed (discrete) time points *t_m_* and *t_m_*_+1_ (*t_m_ < t* ≤ *t_m+1_*) follow an exponential distribution with rate parameter *λ_i_*(***x***(*t*)). In principle, these rate parameters may depend on network or individual characteristics. For simplicity, though, most applications assume all actors have equal opportunities to change their ties in a given period (11,54,55). As such, the rate parameters typically only depend on period, and do not depend on *x* or *i*. That is,

| $\lambda_{i}\left( \boldsymbol{x}(t) \right)=\rho_{m}$ | (1: rate function) |
| --- | --- |

where *ρ_m_* is the rate parameter in a specific time interval *t_m_ < t* ≤ *t_m+1_* for all the actors in the network.

When actor *i* gets the opportunity to change, this actor can engage with *only one* tie variable *x_ij_* (i.e., they are only allowed to change up to one tie variable at each ministep). Where the state of the full network at the start of the ministep is ***x***(*t*), we define ***x***^(±^*^ij^*^)^ as the result of actor *i* choosing a particular tie *x_ij_* to engage with at this ministep. For a given tie *x_ij_* where *i* ≠ *j*, *x_ij_*^(±^*^ij^*^)^ = 1 - *x_ij_* and *x_vw_*^(±^*^ij^*^)^ = *x_vw_* for all (*v,w*) ≠ (*i*,*j*); and for a given tie *x_ij_* where *i* = *j*, *x_ij_*^(±^*^ij^*^)^ *= x_ij_*. Together, these possibilities effectively allow actor *i* to toggle a specific tie at each ministep, either forming a tie when *x_ij_* = 0 → 1, dissolving a tie when *x_ij_* = 1 → 0, or maintaining the status quo. The probability that actor *i* selects *x_ij_* and moves the network to state ***x***^(±^*^ij^*^)^ at each ministep, *p_i_*(***x***^(±^*^ij^*^)^), is then governed by the objective function.

**Objective function*.*** The *objective function* describes the hypothesized mechanisms that drive change and/or persistence in the network, formalizing the hypothesis that actors’ probabilistic choices about which tie *x_ij_* to toggle at each ministep are based on *k* = 1 to *K* predefined structural and/or covariate *effects*. At each ministep, actor *i* makes a choice that can maximize the objective function,

| $f_{i}\left( \boldsymbol{x}^{(\pm ij)} \right\vert\boldsymbol{\beta})=\sum_{k=1}^{K} {\beta_{k}s}_{ik}\left( \boldsymbol{x}^{(\pm ij)} \right)$ | (2: objective function) |
| --- | --- |

where ***x***^(±^*^ij^*^)^ denotes a potential state of the network and *s_ik_*(***x***^(±^*^ij^*^)^) denotes actor *i*’s evaluation of the *k*-th *effect* (*k* = 1, …, *K*) for that potential state. The *β_k_* parameters in ***β*** = (*β_1_, β_2_, …, β_K_*) describe the extent to which each of the specific *effects* contributes to actors’ choices about which ties to toggle, and thus to the evolution of the network over time. In line with a Markov assumption, the *effects* often only depend on the potential state ***x***^(±^*^ij^*^)^, so that actors make decisions that can maximize the objective function regardless of previous state; and in line with the notion of *myopic rationality*, actors make decisions without strategic consideration of how other actors might react (53). Following these assumptions, each *effect* formalizes how a specific network, individual, or dyad characteristic influences actors’ choices about which ties to form, maintain, and dissolve.
