# Supplementary material for: Evolution of networks in virtual reality: A scalable framework for studying social dynamics of small groups
Source: PLoS One. 2026 Aug 11;21(8):e0355470. doi: 10.1371/journal.pone.0355470 (PMC13460626; doi:10.1371/journal.pone.0355470)
Supplement: Supplementary file 2 [file pone.0355470.s004.docx]

**S1 Table**

*Rate parameter estimates from SAOMs*

| Group | Week | Jaccard similarity^#^ | Model 1 | Model 2 | Model 3 |
| --- | --- | --- | --- | --- | --- |
|  |  |  | Actor effects | Actor-partner effects | Dyadic effects |
| 1 | 4 → 5 | **.00** | 9.84 (8.79) | 9.25 (8.42) | 8.80 (7.72) |
|  | 5 → 6 | **.00** | 16.91 (14.49) | 14.18 (11.70) | 11.80 (9.05) |
|  | 6 → 7 | **.00** | **85.38 (92.33)** | 37.75 (43.20) | 27.92 (28.12) |
|  | 7 → 8 | .67 | 0.53 (0.53) | 0.60 (0.60) | 0.65 (0.65) |
| 2 | 4 → 5 | .31 | 3.92 (1.75) | 4.18 (1.92) | 5.18 (2.54) |
|  | 5 → 6 | .15 | **63.31 (69.09)** | 45.26 (43.48) | 47.27 (38.48) |
|  | 6 → 7 | .13 | 6.89 (3.47) | 6.94 (3.60) | 7.53 (4.27) |
|  | 7 → 8 | .43 | 2.46 (1.42) | 2.40 (1.40) | 2.48 (1.43) |
| 3 | 4 → 5 | .10 | 14.12 (9.84) | 13.01 (8.26) | 13.64 (8.41) |
|  | 5 → 6 | .27 | 5.67 (2.84) | 5.41 (2.60) | 6.34 (3.33) |
|  | 6 → 7 | .12 | 10.51 (6.40) | 8.98 (5.19) | 11.26 (7.03) |
|  | 7 → 8 | .22 | 5.20 (2.88) | 5.01 (2.69) | 5.16 (2.92) |
| 4 | 4 → 5 | .20 | 11.07 (8.02) | 9.27 (6.01) | 9.68 (6.36) |
|  | 5 → 6 | .25 | 4.19 (3.01) | 3.92 (2.55) | 3.30 (2.15) |
|  | 6 → 7 | .33 | 3.62 (2.20) | 3.31 (1.97) | 3.27 (1.93) |
|  | 7 → 8 | .17 | 20.84 (16.94) | 32.73 (27.30) | 32.80 (27.23) |
| 5 | 4 → 5 | .33 | 3.39 (2.08) | 3.40 (2.04) | 5.34 (3.54) |
|  | 5 → 6 | .83 | 0.56 (0.55) | 0.55 (0.53) | 0.67 (0.65) |
|  | 6 → 7 | .43 | 2.31 (1.32) | 2.24 (1.26) | 2.73 (1.60) |
|  | 7 → 8 | .60 | 1.58 (1.17) | 1.61 (1.17) | 1.88 (1.37) |
| 6 | 4 → 5 | .12 | 15.43 (10.60) | 19.97 (15.27) | 11.80 (6.47) |
|  | 5 → 6 | .32 | 6.12 (2.59) | 6.21 (2.62) | 5.62 (2.30) |
|  | 6 → 7 | .29 | 5.02 (1.77) | 5.64 (2.15) | 7.02 (3.19) |
|  | 7 → 8 | .11 | 10.98 (9.68) | 16.57 (17.17) | 18.22 (16.37) |
| 7 | 4 → 5 | .12 | 7.45 (3.81) | 7.15 (3.43) | 19.75 (16.26) |
|  | 5 → 6 | .43 | 2.43 (1.04) | 2.43 (1.00) | 2.82 (1.19) |
|  | 6 → 7 | .14 | 17.95 (15.40) | 16.67 (13.52) | **249.50 (250.80)** |
|  | 7 → 8 | .25 | 4.89 (2.80) | 5.20 (2.86) | 4.73 (2.31) |
| 8 | 4 → 5 | .11 | 13.18 (7.13) | 14.05 (7.51) | 12.41 (6.18) |
|  | 5 → 6 | .25 | 11.83 (8.24) | 11.75 (7.41) | 10.02 (5.37) |
|  | 6 → 7 | .25 | 7.01 (3.22) | 7.12 (3.23) | 6.99 (3.17) |
|  | 7 → 8 | .22 | 6.19 (2.91) | 6.25 (3.03) | 6.33 (3.12) |

*Note.* Rate parameter estimates with standard errors in parentheses. Rates indicate the expected frequencies of change in each network transition. Groups with extremely high rate parameters (larger than 50 in **bold**) should be treated with caution (Ripley et al., 2024, pp. 123-124).

^#^Jaccard similarity (stability) measure between the successive networks = *N*_11_ / (*N*_01_ + *N*_10_ + *N*_00_), where *N_pq_* is the number of tie variables with value *p* in one wave and value *q* in the next wave. Low Jaccard values (lower than .1 in **bold**) should be treated with caution (Ripley et al., 2024, pp. 19-20).
