# Supplementary material for: Evolution of networks in virtual reality: A scalable framework for studying social dynamics of small groups
Source: PLoS One. 2026 Aug 11;21(8):e0355470. doi: 10.1371/journal.pone.0355470 (PMC13460626; doi:10.1371/journal.pone.0355470)
Supplement: Supplementary file 3 [file pone.0355470.s005.docx]

**S2 Table**

*Results from SAOMs examining how structural, individual-level covariate, and dyad-level covariate effects contribute to network change: Using 3.65-meter threshold for tie presence*

|  | Model 1 | Model 2 | Model 3 |
| --- | --- | --- | --- |
| Parameter | Actor effects | Actor-partner effects | Dyadic effects |
| *Structural effects* |  |  |  |
| Out-degree | **-0.847*** (0.122)** | **-0.804*** (0.173)** | **-0.683*** (0.177)** |
| GWESP | **0.922*** (0.113)** | **0.945*** (0.113)** | **0.881*** (0.118)** |
| *Individual-level covariate effects* |  |  |  |
| Time | -0.040 (0.034) | -0.044 (0.033) | -0.053 (0.035) |
| Prior VR use | -0.109 (0.123) | -0.106 (0.141) | -0.147 (0.141) |
| Prior acquaintance | 0.020 (0.125) | 0.077 (0.127) | -0.158 (0.146) |
| Group identification | -0.034 (0.062) | -0.084 (0.061) | -0.102 (0.062) |
| Prior VR use imbalance^a^ |  | 0.211 (0.119) | 0.174 (0.119) |
| Prior acquaintance imbalance^a^ |  | -0.162 (0.128) | -0.049 (0.124) |
| Group identification imbalance^a^ |  | **-0.166** (0.064)** | **-0.163* (0.066)** |
| Same gender |  | 0.156 (0.098) | 0.011 (0.108) |
| Same ethnicity |  | -0.112 (0.129) | -0.177 (0.130) |
| *Dyad-level covariate effects* |  |  |  |
| Familiarity |  |  | **0.356*** (0.067)** |
| Friendship |  |  | -0.049 (0.063) |
| Chi-square statistic (*df*)^b^ |  | **16.070*** (5)** | **33.703*** (2)** |
| Overall maximum convergence ratio | .066 | .111 | .116 |
| Absolute maximum of *t*-statistics | .033 | .062 | .050 |

*Note*. GWESP = geometrically weighted shared partners. Multigroup models fit by the conditional Method of Moments estimation with 3,000 iterations in the third phase, using the “RSiena” package (version 1.4.7). Model convergence is obtained when the overall maximum convergence ratio < .20 and the absolute value of individual *t*-statistics < .10. Parameter estimates with standard errors in parentheses. Statistically significant parameters indicated in **bold** font. **p* < .05. ***p* < .01. ****p* < .001. ^a^Imbalance is measured by the absolute sum of differences between a focal actor and its partners.

^b^Tests null hypothesis that newly added parameters are zero.
