# Supplementary material for: Evolution of networks in virtual reality: A scalable framework for studying social dynamics of small groups
Source: PLoS One. 2026 Aug 11;21(8):e0355470. doi: 10.1371/journal.pone.0355470 (PMC13460626; doi:10.1371/journal.pone.0355470)
Supplement: Supplementary file 4 [file pone.0355470.s006.docx]

**S3 Table**

*Descriptive statistics of observed weekly group networks*

|  |  | Number of Distinct Ties (Density) | | | | |
| --- | --- | --- | --- | --- | --- | --- |
|  | *N* | Week 4 | Week 5 | Week 6 | Week 7 | Week 8 |
| Group 1 | 10 | 6 (0.13) | 6 (0.13) | 5 (0.11) | 6 (0.13) | 4 (0.09) |
| Group 2 | 12 | 11 (0.17) | 13 (0.20) | 15 (0.23) | 5 (0.08) | 8 (0.12) |
| Group 3 | 14 | 9 (0.10) | 7 (0.08) | 11 (0.12) | 10 (0.11) | 7 (0.08) |
| Group 4 | 9 | 12 (0.33) | 9 (0.25) | 4 (0.11) | 8 (0.22) | 6 (0.17) |
| Group 5 | 12 | 11 (0.17) | 7 (0.11) | 6 (0.09) | 5 (0.08) | 5 (0.08) |
| Group 6 | 14 | 12 (0.13) | 12 (0.13) | 23 (0.25) | 15 (0.16) | 12 (0.13) |
| Group 7 | 14 | 16 (0.18) | 10 (0.11) | 13 (0.14) | 9 (0.10) | 11 (0.12) |
| Group 8 | 14 | 15 (0.16) | 12 (0.13) | 16 (0.18) | 9 (0.10) | 8 (0.09) |

*Note*. *N* = number of actors. Self-ties are not permitted in symmetric networks. Network density is calculated as the number of distinct ties divided by *N*(*N*-1).
