# Supplementary material for: Evolution of networks in virtual reality: A scalable framework for studying social dynamics of small groups
Source: PLoS One. 2026 Aug 11;21(8):e0355470. doi: 10.1371/journal.pone.0355470 (PMC13460626; doi:10.1371/journal.pone.0355470)
Supplement: Supplementary file 5 [file pone.0355470.s007.html]

Supplemental R Scripts: VR Network


- Supplemental R Scripts: VR Network

- Load
  Libraries
- Load Data
  - (1) Interpersonal
    distance
  - (2) Individual
    difference measures
  - (3) Dyadic relationship
    measures
  - (4) Time
- Fit Stochastic
  Actor-Oriented Models
  - Stage 1:
    Design of longitudinal network
  - Stage 2: Model
    definition
    - Model 1: Actor effects
    - Model 2: Actor-partner
      effects
    - Model 3: Dyadic
      effects
  - Stage 3: Model
    estimation and selection
- SAOM
  Results
  - Model 1: Actor
    effects
  - Model 2:
    Actor-partner effects
  - Model 3: Dyadic
    effects
- R
  Environment

- 2026-06-17

# Supplemental R Scripts: VR Network

# Load Libraries

```
library(tidyverse) # for data manipulation
library(psych) # for descriptive statistics
library(RSiena) # for network analysis
```

# Load Data

## (1) Interpersonal distance

Distances (`distance`) were calculated for each pair of
two participants (`participant_id_x` and
`participant_id_y`) in each weekly (`week`)
discussion section (`section`).

```
dta_long %>% select(week:distance) %>% head()
```

```
## # A tibble: 6 × 5
##    week section participant_id_x participant_id_y distance
##   <int>   <int> <chr>            <chr>               <dbl>
## 1     4       1 79fc7927         7b34366b           11    
## 2     4       1 79fc7927         7caffbba            1    
## 3     4       1 79fc7927         a7afa4c9            0.667
## 4     4       1 79fc7927         ca730476           11.7  
## 5     4       1 79fc7927         e2dcea3c            1.67 
## 6     4       1 7b34366b         7caffbba           11.3
```

Calculate the number of nodes per section.

```
N <- dta_long %>%
  select(section, node1, node2) %>% 
  pivot_longer(cols = node1:node2) %>% 
  group_by(section) %>% 
  summarize(N = n_distinct(value)) %>% 
  pull(N)
print(N)
```

```
## [1] 10 12 14  9 12 14 14 14
```

Calculate weekly network densities per section.

```
dta_long_comp <- dta_long %>% 
  filter(distance < 4) # filter distance < 4 ties only
table(dta_long_comp$section, dta_long_comp$week)*2 / (N*(N-1))
```

```
##    
##              4          5          6          7          8
##   1 0.13333333 0.13333333 0.11111111 0.13333333 0.08888889
##   2 0.16666667 0.19696970 0.22727273 0.07575758 0.12121212
##   3 0.09890110 0.07692308 0.12087912 0.10989011 0.07692308
##   4 0.33333333 0.25000000 0.11111111 0.22222222 0.16666667
##   5 0.16666667 0.10606061 0.09090909 0.07575758 0.07575758
##   6 0.13186813 0.13186813 0.25274725 0.16483516 0.13186813
##   7 0.17582418 0.10989011 0.14285714 0.09890110 0.12087912
##   8 0.16483516 0.13186813 0.17582418 0.09890110 0.08791209
```

```
apply(table(dta_long_comp$section, dta_long_comp$week)*2 / (N*(N-1)),2,mean)
```

```
##         4         5         6         7         8 
## 0.1714286 0.1421141 0.1540890 0.1224498 0.1087635
```

```
apply(table(dta_long_comp$section, dta_long_comp$week)*2 / (N*(N-1)),2,sd)
```

```
##          4          5          6          7          8 
## 0.07030323 0.05545098 0.05913263 0.04999523 0.03189568
```

## (2) Individual difference measures

Prior VR use.

```
qst_wide %>% count(priorVR_raw, priorVR)
```

```
## # A tibble: 5 × 3
##   priorVR_raw priorVR     n
##         <dbl>   <dbl> <int>
## 1           0       0    42
## 2           1       1     6
## 3           2       1     6
## 4           3       1     7
## 5           4       1    20
```

Prior acquaintance.

```
qst_wide %>% count(priorAcq_raw, priorAcq)
```

```
## # A tibble: 7 × 3
##   priorAcq_raw priorAcq     n
##          <dbl>    <dbl> <int>
## 1            0        0    38
## 2            1        1    13
## 3            2        1    12
## 4            3        1     1
## 5            4        1     2
## 6            5        1     2
## 7           NA       NA    13
```

Group identification.

```
qst_wide %>% 
  summarize(groupIdn_rawM = mean(groupIdn_raw, na.rm=T), 
            groupIdn_rawSD = sd(groupIdn_raw, na.rm=T))
```

```
## # A tibble: 1 × 2
##   groupIdn_rawM groupIdn_rawSD
##           <dbl>          <dbl>
## 1          5.35          0.966
```

Gender.

```
qst_wide %>% count(gender)
```

```
## # A tibble: 5 × 2
##   gender     n
##    <dbl> <int>
## 1      0    47
## 2      1    30
## 3      3     2
## 4      4     1
## 5     NA     1
```

Ethnicity.

```
qst_wide %>% count(ethnicity)
```

```
## # A tibble: 8 × 2
##   ethnicity     n
##       <dbl> <int>
## 1         0     3
## 2         1    11
## 3         2    30
## 4         3     9
## 5         5     1
## 6         7    21
## 7         8     5
## 8        NA     1
```

Describe variables after preprocessing.

```
coCovars <- c("priorVR", "priorAcq", "groupIdn", "gender", "ethnicity")
qst_wide %>%
  select(all_of(coCovars)) %>%
  describe()
```

```
##           vars  n mean   sd median trimmed  mad   min  max range  skew kurtosis
## priorVR      1 81 0.48 0.50   0.00    0.48 0.00  0.00 1.00     1  0.07    -2.02
## priorAcq     2 68 0.44 0.50   0.00    0.43 0.00  0.00 1.00     1  0.23    -1.97
## groupIdn     3 79 0.00 0.97   0.28    0.03 1.11 -2.35 1.65     4 -0.38    -0.71
## gender       4 80 0.50 0.75   0.00    0.39 0.00  0.00 4.00     4  2.17     6.39
## ethnicity    5 80 3.62 2.60   2.00    3.50 1.48  0.00 8.00     8  0.54    -1.41
##             se
## priorVR   0.06
## priorAcq  0.06
## groupIdn  0.11
## gender    0.08
## ethnicity 0.29
```

## (3) Dyadic relationship measures

Familiarity.

```
pair_long %>%
  summarize(familiarity_rawM = mean(familiarity_raw,na.rm=T),
            familiarity_rawSD = sd(familiarity_raw,na.rm=T))
```

```
## # A tibble: 1 × 2
##   familiarity_rawM familiarity_rawSD
##              <dbl>             <dbl>
## 1             2.13              1.16
```

Friendship.

```
pair_long %>%
  summarize(friendship_rawM = mean(friendship_raw,na.rm=T),
            friendship_rawSD = sd(friendship_raw,na.rm=T))
```

```
## # A tibble: 1 × 2
##   friendship_rawM friendship_rawSD
##             <dbl>            <dbl>
## 1            4.99             1.27
```

Describe variables after preprocessing.

```
pair_long %>%
  select(familiarity, friendship) %>%
  describe()
```

```
##             vars   n mean   sd median trimmed  mad   min  max range  skew
## familiarity    1 704    0 1.16  -0.13   -0.17 1.48 -1.13 3.87     5  0.95
## friendship     2 714    0 1.27   0.01   -0.01 1.48 -3.99 3.01     7 -0.28
##             kurtosis   se
## familiarity     0.26 0.04
## friendship      0.21 0.05
```

## (4) Time

```
qst_long %>% count(week, time)
```

```
## # A tibble: 5 × 3
##    week  time     n
##   <dbl> <dbl> <int>
## 1     4    -2    81
## 2     5    -1    81
## 3     6     0    81
## 4     7     1    81
## 5     8     2    81
```

Describe variables after preprocessing.

```
varCovars <- c("time")
qst_long %>%
  select(all_of(varCovars)) %>%
  describe()
```

```
##      vars   n mean   sd median trimmed  mad min max range skew kurtosis   se
## time    1 405    0 1.42      0       0 1.48  -2   2     4    0    -1.31 0.07
```

# Fit Stochastic Actor-Oriented Models

We demonstrate how Stochastic Actor-Oriented Models (SAOMs) methods
can be used to study small group networks in VR classrooms. The analysis
is housed in three stages.

## Stage 1: Design of longitudinal network

```
# Create node ids
dta_long_ids <- sort(unique(c(dta_long$node1, dta_long$node2)))
dta_long2 <- dta_long %>% 
  left_join(dta_long_ids %>% enframe(name="Var1"), by=c("node1"="value")) %>% 
  left_join(dta_long_ids %>% enframe(name="Var2"), by=c("node2"="value"))

# Save the objects using subscripts
M <- dta_long2 %>% distinct(week) %>% nrow()
full_sec <- 1:8
S <- length(full_sec)

# Create input data for SAOMs
create_data <- function(s){
  # Subset section=s participants
  dta_long3 <- dta_long2 %>% filter(section==s)
  dta_long3_ids <- sort(unique(c(dta_long3$Var1, dta_long3$Var2)))
  n <- length(dta_long3_ids) # number of participants in each section
  dta_long3_lgs <- bind_rows(
    dta_long3 %>% distinct(login=login.x, Var=Var1),
    dta_long3 %>% distinct(login=login.y, Var=Var2)) %>% 
    distinct()
  
  # Define distance matrices
  dist_mat <- dta_long3 %>% 
    right_join(dta_long3 %>% distinct(week,section), by=c("week","section")) %>% 
    mutate(distance = ifelse(distance < 4.00, 1, 0)) %>% # set threshold 
    group_by(Var1,Var2) %>% 
    split(.$week) %>% 
    map(. %>%
          right_join(expand.grid(dta_long3_ids, dta_long3_ids) %>% as_tibble(),
                         by = c("Var1", "Var2")) %>% 
          select(Var1, Var2, distance) %>% arrange(Var2) %>%
          pivot_wider(names_from=Var2, values_from=distance) %>% 
          ungroup() %>%
          column_to_rownames("Var1") %>% 
          as.matrix() %>% 
          Matrix::forceSymmetric(., uplo="U") %>% # make it symmetric
          as.matrix())
  for(t in 1:M){
    diag(dist_mat[[t]]) <- NA # coerce within-person distances to be NA
    dist_mat[[t]][is.infinite(dist_mat[[t]])] <- NA
  }
  # Define dependent binary adjacency matrices
  dep <- dist_mat %>% 
    unlist() %>% array(., dim=c(n,n,M)) # diagonals are always NA
  siena_dep <- sienaDependent(dep, allowOnly=FALSE)
  
  # Add time-invariant individual difference measures
  qVarsw <- qst_wide %>%
    filter(login %in% dta_long3_lgs$login) %>% 
    right_join(dta_long3_lgs, by="login")
  qcoCovars <- qVarsw %>% select(Var, all_of(coCovars)) %>% distinct() %>%
    arrange(Var) %>% select(-Var) %>% data.frame()
  for (i in coCovars) {
    assign(eval(i), coCovar(qcoCovars[[i]], centered=FALSE)) # centered already
  }
  
  # Add time-varying individual difference measures
  qVarsl <- qst_long %>%
    filter(login %in% dta_long3_lgs$login) %>%
    right_join(dta_long3_lgs, by="login")
  for (j in c(varCovars)) {
    qVarsl1 <- qVarsl %>% rename("j"=all_of(j)) %>%
      select(Var,week,"j") %>%
      arrange(Var,week) %>%
      drop_na() %>%
      pivot_wider(names_from=week, values_from=j, names_prefix="w") %>%
      select(Var,paste0("w",4:8)) %>%
      right_join(dta_long3_lgs, by="Var") %>%
      arrange(Var) %>% select(-Var,-login) %>% data.frame() %>% data.matrix()
    assign(eval(j), varCovar(qVarsl1, centered=FALSE)) # centered already
  }
  
  # Add dyadic relationship measures
  pair_long2 <- pair_long %>%
    filter((login.x %in% dta_long3_lgs$login)|(login.y %in% dta_long3_lgs$login))
  pair_long2_ids <- sort(unique(c(pair_long2$login.x, pair_long2$login.y)))
  pair_mat_fam <- pair_long2 %>%
    select(login.x, login.y, familiarity) %>%
    left_join(dta_long3_lgs, by=c("login.x"="login")) %>% rename(Var1=Var) %>% 
    left_join(dta_long3_lgs, by=c("login.y"="login")) %>% rename(Var2=Var) %>% 
    select(Var1, Var2, familiarity) %>% 
    right_join(expand.grid(dta_long3_ids, dta_long3_ids) %>% as_tibble(),
               by=c("Var1", "Var2")) %>% 
    arrange(Var1,Var2) %>% 
    pivot_wider(names_from=Var2, values_from=familiarity) %>% 
    select(-Var1) %>% 
    as.matrix()
  pair_mat_frd <- pair_long2 %>%
    select(login.x, login.y, friendship) %>%
    left_join(dta_long3_lgs, by=c("login.x"="login")) %>% rename(Var1=Var) %>% 
    left_join(dta_long3_lgs, by=c("login.y"="login")) %>% rename(Var2=Var) %>% 
    select(Var1, Var2, friendship) %>% 
    right_join(expand.grid(dta_long3_ids, dta_long3_ids) %>% as_tibble(),
               by=c("Var1", "Var2")) %>% 
    arrange(Var1,Var2) %>% 
    pivot_wider(names_from=Var2, values_from=friendship) %>% 
    select(-Var1) %>% 
    as.matrix()
  siena_fam <- coDyadCovar(pair_mat_fam, centered=FALSE) # centered already
  siena_frd <- coDyadCovar(pair_mat_frd, centered=FALSE) # centered already
  
  # Combine 
  siena_data <- sienaDataCreate(y=siena_dep, 
                                priorVR, priorAcq, groupIdn, time,
                                gender, ethnicity,
                                familiarity=siena_fam, friendship=siena_frd)
  siena_data
}
siena_datalist <- map(full_sec,create_data)
siena_data <- sienaGroupCreate(siena_datalist)
```

The threshold = 4.00 (meter) was median distance across all
participant pairs.

```
dta %>%
  filter(distance < 15) %>%
  ggplot(aes(x = distance)) +
  geom_histogram(binwidth = 0.1, boundary = 0) +
  geom_vline(xintercept=4.00,linetype="dashed",color="blue")+
  theme_bw()+
  labs(x="Interpersonal Distance (unit = meter)",y="Frequency")
```

## Stage 2: Model definition

### Model 1: Actor effects

```
siena_eff1 <- getEffects(siena_data)
siena_eff1 <- includeEffects(siena_eff1, gwesp)
siena_eff1 <- includeEffects(siena_eff1, egoX, interaction1="priorVR")
siena_eff1 <- includeEffects(siena_eff1, egoX, interaction1="priorAcq")
siena_eff1 <- includeEffects(siena_eff1, egoX, interaction1="groupIdn")
siena_eff1 <- includeEffects(siena_eff1, egoX, interaction1="time")
```

### Model 2: Actor-partner effects

```
siena_eff2 <- includeEffects(siena_eff1, absDiffX, interaction1="priorVR")
siena_eff2 <- includeEffects(siena_eff2, absDiffX, interaction1="priorAcq")
siena_eff2 <- includeEffects(siena_eff2, absDiffX, interaction1="groupIdn")
siena_eff2 <- includeEffects(siena_eff2, sameX, interaction1="gender")
siena_eff2 <- includeEffects(siena_eff2, sameX, interaction1="ethnicity")
```

### Model 3: Dyadic effects

```
siena_eff3 <- includeEffects(siena_eff2, X, interaction1="familiarity")
siena_eff3 <- includeEffects(siena_eff3, X, interaction1="friendship")
```

## Stage 3: Model estimation and selection

Define function for fitting models.

```
fit_model <- function(siena_data, siena_eff){
  siena_alg <- sienaAlgorithmCreate(cond = TRUE, n3 = 3000, modelType = c(y=3),
                                    seed = 12345)
  siena_res <- siena07(siena_alg, data = siena_data, effects = siena_eff, 
                       batch = TRUE, useCluster = TRUE, nbrNodes = 6, 
                       returnDeps = TRUE)
  return(siena_res)
}
```

Define function for summarizing results.

```
tidy_output <- function(siena_res){
  param <- siena_res$effects$effectName
  est <- siena_res$theta
  se <- sqrt(diag(siena_res$covtheta))
  tstat <- est/se
  pval <- 2*pnorm(abs(tstat), lower.tail = FALSE)
  table2 <- tibble(parameter = param, estimate = est, SE = se,
                   pvalue = pval, tconv = siena_res$tconv) %>% 
    mutate(star = cut(pvalue,
                      breaks = c(-Inf,.001,.01,.05,1),
                      labels = c("***","**","*",""),
                      include.lowest = T),
           print = str_c(round(estimate,3),star),
           exp = exp(estimate) %>% round(3),
           SE = round(SE,3),
           pvalue = round(pvalue,3),
           tconv = round(tconv,3)) %>%
    select(parameter, print, exp, SE, pvalue, tconv)
  return(table2)
}
```

# SAOM Results

## Model 1: Actor effects

```
siena_res1 <- fit_model(siena_data, siena_eff1)
```

```
## If you use this algorithm object, siena07 will create/use an output file Siena.txt .
## 
## Start phase 0 
## theta: -0.506  0.000  0.000  0.000  0.000  0.000 
## 
## Start phase 1 
## Phase 1 Iteration 1 Progress: 0%
## Phase 1 Iteration 25 Progress: 0%
## theta: -0.59773  0.65306 -0.00252  0.03608 -0.16677 -0.05873 
## 
## Start phase 2.1
## Phase 2 Subphase 1 Iteration 1 Progress: 8%
## Phase 2 Subphase 1 Iteration 2 Progress: 8%
## theta -0.67349  0.88751 -0.00484 -0.06201 -0.11465 -0.05469 
## ac  0.2412 -0.2166 -0.0478  0.4300 -0.5006  0.2070 
## Phase 2 Subphase 1 Iteration 3 Progress: 8%
## Phase 2 Subphase 1 Iteration 4 Progress: 8%
## theta -0.7786  0.9514 -0.0522 -0.1211  0.0897 -0.0148 
## ac -0.727 -0.816 -0.972 -0.715 -0.770  0.465 
## Phase 2 Subphase 1 Iteration 5 Progress: 8%
## Phase 2 Subphase 1 Iteration 6 Progress: 8%
## theta -0.8062  0.8647 -0.0540 -0.1068  0.0670 -0.0625 
## ac -0.890 -0.910 -1.144 -0.926 -0.968  0.268 
## Phase 2 Subphase 1 Iteration 7 Progress: 8%
## Phase 2 Subphase 1 Iteration 8 Progress: 8%
## theta -0.8369  1.1729 -0.0342 -0.0980 -0.0619 -0.0575 
## ac -0.454 -0.416 -0.812 -0.436 -0.566  0.206 
## Phase 2 Subphase 1 Iteration 9 Progress: 8%
## Phase 2 Subphase 1 Iteration 10 Progress: 8%
## theta -0.8114  1.0802 -0.0668 -0.0889 -0.0146 -0.0618 
## ac -0.6026 -0.5131 -0.7843 -0.5740 -0.6578  0.0406 
## theta -0.7668  0.7028 -0.0490 -0.1238  0.0251 -0.0310 
## ac -0.644 -0.553 -0.733 -0.627 -0.693 -0.304 
## theta: -0.7668  0.7028 -0.0490 -0.1238  0.0251 -0.0310 
## 
## Start phase 2.2
## Phase 2 Subphase 2 Iteration 1 Progress: 13%
## Phase 2 Subphase 2 Iteration 2 Progress: 13%
## Phase 2 Subphase 2 Iteration 3 Progress: 13%
## Phase 2 Subphase 2 Iteration 4 Progress: 13%
## Phase 2 Subphase 2 Iteration 5 Progress: 13%
## Phase 2 Subphase 2 Iteration 6 Progress: 13%
## Phase 2 Subphase 2 Iteration 7 Progress: 13%
## Phase 2 Subphase 2 Iteration 8 Progress: 13%
## Phase 2 Subphase 2 Iteration 9 Progress: 13%
## Phase 2 Subphase 2 Iteration 10 Progress: 13%
## theta -0.7704  0.8251 -0.0424 -0.1220 -0.0187 -0.0265 
## ac -0.732 -0.694 -0.732 -0.744 -0.760 -0.794 
## theta: -0.7704  0.8251 -0.0424 -0.1220 -0.0187 -0.0265 
## 
## Start phase 2.3
## Phase 2 Subphase 3 Iteration 1 Progress: 18%
## Phase 2 Subphase 3 Iteration 2 Progress: 18%
## Phase 2 Subphase 3 Iteration 3 Progress: 18%
## Phase 2 Subphase 3 Iteration 4 Progress: 18%
## Phase 2 Subphase 3 Iteration 5 Progress: 18%
## Phase 2 Subphase 3 Iteration 6 Progress: 18%
## Phase 2 Subphase 3 Iteration 7 Progress: 18%
## Phase 2 Subphase 3 Iteration 8 Progress: 18%
## Phase 2 Subphase 3 Iteration 9 Progress: 18%
## Phase 2 Subphase 3 Iteration 10 Progress: 18%
## theta -0.83890  0.89808 -0.03633 -0.09563  0.00131 -0.02646 
## ac -0.61005 -0.70385 -0.00429 -0.74605 -0.68277 -0.48736 
## theta: -0.83890  0.89808 -0.03633 -0.09563  0.00131 -0.02646 
## 
## Start phase 2.4
## Phase 2 Subphase 4 Iteration 1 Progress: 24%
## Phase 2 Subphase 4 Iteration 2 Progress: 24%
## Phase 2 Subphase 4 Iteration 3 Progress: 24%
## Phase 2 Subphase 4 Iteration 4 Progress: 24%
## Phase 2 Subphase 4 Iteration 5 Progress: 24%
## Phase 2 Subphase 4 Iteration 6 Progress: 24%
## Phase 2 Subphase 4 Iteration 7 Progress: 24%
## Phase 2 Subphase 4 Iteration 8 Progress: 24%
## Phase 2 Subphase 4 Iteration 9 Progress: 24%
## Phase 2 Subphase 4 Iteration 10 Progress: 24%
## theta -0.83495  0.89766 -0.03638 -0.09792  0.00664 -0.02193 
## ac -0.2364 -0.2877 -0.2618 -0.2858 -0.2068 -0.0602 
## theta: -0.83495  0.89766 -0.03638 -0.09792  0.00664 -0.02193 
## 
## Start phase 3 
## Phase 3 Iteration 1 Progress 33%
## Phase 3 Iteration 601 Progress 46%
## Phase 3 Iteration 1201 Progress 60%
## Phase 3 Iteration 1801 Progress 73%
## Phase 3 Iteration 2401 Progress 87%
```

```
tidy_output(siena_res1)
```

```
## # A tibble: 6 × 6
##   parameter        print       exp    SE pvalue  tconv
##   <chr>            <chr>     <dbl> <dbl>  <dbl>  <dbl>
## 1 degree (density) -0.835*** 0.434 0.123  0      0.034
## 2 GWESP (69)       0.898***  2.45  0.113  0      0.024
## 3 time ego         -0.036    0.964 0.034  0.286 -0.023
## 4 priorVR ego      -0.098    0.907 0.117  0.405  0.068
## 5 priorAcq ego     0.007     1.01  0.124  0.957  0.002
## 6 groupIdn ego     -0.022    0.978 0.06   0.715 -0.001
```

## Model 2: Actor-partner effects

```
siena_res2 <- fit_model(siena_data, siena_eff2)
```

```
## If you use this algorithm object, siena07 will create/use an output file Siena.txt .
## 
## Start phase 0 
## theta: -0.506  0.000  0.000  0.000  0.000  0.000  0.000  0.000  0.000  0.000  0.000 
## 
## Start phase 1 
## Phase 1 Iteration 1 Progress: 0%
## Phase 1 Iteration 25 Progress: 0%
## theta: -0.51077  0.56825 -0.00135  0.08046  0.06128 -0.06034  0.00297 -0.05832 -0.11304  0.03808 -0.04692 
## 
## Start phase 2.1
## Phase 2 Subphase 1 Iteration 1 Progress: 13%
## Phase 2 Subphase 1 Iteration 2 Progress: 13%
## theta -0.5481  0.7548 -0.0438 -0.0150  0.0831 -0.1042 -0.0287 -0.0505 -0.1336  0.0596 -0.1043 
## ac  0.737 -1.193 -0.647  0.946  0.791  1.142  0.695  0.519  0.764  0.913  1.231 
## Phase 2 Subphase 1 Iteration 3 Progress: 13%
## Phase 2 Subphase 1 Iteration 4 Progress: 13%
## theta -0.6403  0.7956 -0.0538 -0.2219  0.1512  0.0303 -0.2049 -0.0505 -0.1721  0.1227 -0.1109 
## ac -0.1307 -0.2288 -0.5901  0.0888 -0.0565 -0.0110  0.1347  0.9938  0.0453 -0.1884  0.4273 
## Phase 2 Subphase 1 Iteration 5 Progress: 13%
## Phase 2 Subphase 1 Iteration 6 Progress: 13%
## theta -0.6928  0.3679 -0.0833 -0.2680  0.1719  0.1787 -0.3136 -0.0141 -0.1854  0.1734 -0.0833 
## ac -1.325 -1.613 -1.326 -1.073 -1.225 -1.331 -1.012 -0.316 -1.383 -1.295 -1.105 
## Phase 2 Subphase 1 Iteration 7 Progress: 13%
## Phase 2 Subphase 1 Iteration 8 Progress: 13%
## theta -0.7061  0.2645 -0.0912 -0.2672  0.1963  0.2817 -0.3027 -0.0317 -0.2081  0.1841 -0.0578 
## ac -1.240 -1.547 -1.235 -1.140 -1.232 -1.236 -0.946 -0.632 -1.203 -1.270 -1.182 
## Phase 2 Subphase 1 Iteration 9 Progress: 13%
## Phase 2 Subphase 1 Iteration 10 Progress: 13%
## theta -0.7002  0.2140 -0.0921 -0.3251  0.1967  0.2276 -0.2282 -0.0682 -0.1849  0.1841 -0.0739 
## ac -1.190 -1.436 -1.171 -1.093 -1.181 -1.221 -0.938 -0.628 -1.107 -1.232 -1.222 
## theta -0.6587  0.7429 -0.0606 -0.1621  0.1880  0.1173 -0.2029 -0.0850 -0.2034  0.1511 -0.0929 
## ac -1.160 -1.337 -1.236 -1.074 -1.173 -1.234 -0.974 -0.752 -1.099 -1.144 -1.084 
## theta: -0.6587  0.7429 -0.0606 -0.1621  0.1880  0.1173 -0.2029 -0.0850 -0.2034  0.1511 -0.0929 
## 
## Start phase 2.2
## Phase 2 Subphase 2 Iteration 1 Progress: 17%
## Phase 2 Subphase 2 Iteration 2 Progress: 17%
## Phase 2 Subphase 2 Iteration 3 Progress: 17%
## Phase 2 Subphase 2 Iteration 4 Progress: 17%
## Phase 2 Subphase 2 Iteration 5 Progress: 17%
## Phase 2 Subphase 2 Iteration 6 Progress: 17%
## Phase 2 Subphase 2 Iteration 7 Progress: 17%
## Phase 2 Subphase 2 Iteration 8 Progress: 17%
## Phase 2 Subphase 2 Iteration 9 Progress: 17%
## Phase 2 Subphase 2 Iteration 10 Progress: 17%
## theta -0.6763  0.8086 -0.0498 -0.1177  0.1657  0.0834 -0.1869 -0.0830 -0.1942  0.1315 -0.0609 
## ac -0.919 -0.881 -0.854 -0.908 -0.917 -0.905 -0.890 -0.874 -0.894 -0.915 -0.905 
## theta: -0.6763  0.8086 -0.0498 -0.1177  0.1657  0.0834 -0.1869 -0.0830 -0.1942  0.1315 -0.0609 
## 
## Start phase 2.3
## Phase 2 Subphase 3 Iteration 1 Progress: 22%
## Phase 2 Subphase 3 Iteration 2 Progress: 22%
## Phase 2 Subphase 3 Iteration 3 Progress: 22%
## Phase 2 Subphase 3 Iteration 4 Progress: 22%
## Phase 2 Subphase 3 Iteration 5 Progress: 22%
## Phase 2 Subphase 3 Iteration 6 Progress: 22%
## Phase 2 Subphase 3 Iteration 7 Progress: 22%
## Phase 2 Subphase 3 Iteration 8 Progress: 22%
## Phase 2 Subphase 3 Iteration 9 Progress: 22%
## Phase 2 Subphase 3 Iteration 10 Progress: 22%
## theta -0.7491  0.9173 -0.0365 -0.1075  0.1802  0.0681 -0.1819 -0.0716 -0.1727  0.1322 -0.0929 
## ac -0.642 -0.714 -0.806 -0.511 -0.493 -0.214 -0.529 -0.162 -0.523 -0.282 -0.619 
## theta: -0.7491  0.9173 -0.0365 -0.1075  0.1802  0.0681 -0.1819 -0.0716 -0.1727  0.1322 -0.0929 
## 
## Start phase 2.4
## Phase 2 Subphase 4 Iteration 1 Progress: 28%
## Phase 2 Subphase 4 Iteration 2 Progress: 28%
## Phase 2 Subphase 4 Iteration 3 Progress: 28%
## Phase 2 Subphase 4 Iteration 4 Progress: 28%
## Phase 2 Subphase 4 Iteration 5 Progress: 28%
## Phase 2 Subphase 4 Iteration 6 Progress: 28%
## Phase 2 Subphase 4 Iteration 7 Progress: 28%
## Phase 2 Subphase 4 Iteration 8 Progress: 28%
## Phase 2 Subphase 4 Iteration 9 Progress: 28%
## Phase 2 Subphase 4 Iteration 10 Progress: 28%
## Phase 2 Subphase 4 Iteration 100 Progress: 30%
## theta -0.7501  0.9234 -0.0322 -0.1138  0.1818  0.0866 -0.1674 -0.0704 -0.1692  0.1102 -0.1105 
## ac -0.2188 -0.2189 -0.2117 -0.2608 -0.1829 -0.1269 -0.1386 -0.1173 -0.0553 -0.2185 -0.1921 
## theta -0.7567  0.9166 -0.0358 -0.0989  0.1828  0.0807 -0.1849 -0.0754 -0.1736  0.1388 -0.0864 
## ac -0.3323 -0.2841 -0.1990 -0.3340 -0.4017 -0.2796 -0.2773 -0.2223 -0.2049 -0.2539 -0.0328 
## theta: -0.7567  0.9166 -0.0358 -0.0989  0.1828  0.0807 -0.1849 -0.0754 -0.1736  0.1388 -0.0864 
## 
## Start phase 3 
## Phase 3 Iteration 1 Progress 36%
## Phase 3 Iteration 301 Progress 43%
## Phase 3 Iteration 601 Progress 49%
## Phase 3 Iteration 901 Progress 56%
## Phase 3 Iteration 1201 Progress 62%
## Phase 3 Iteration 1501 Progress 68%
## Phase 3 Iteration 1801 Progress 75%
## Phase 3 Iteration 2101 Progress 81%
## Phase 3 Iteration 2401 Progress 87%
## Phase 3 Iteration 2701 Progress 94%
```

```
tidy_output(siena_res2)
```

```
## # A tibble: 11 × 6
##    parameter                print       exp    SE pvalue  tconv
##    <chr>                    <chr>     <dbl> <dbl>  <dbl>  <dbl>
##  1 degree (density)         -0.757*** 0.469 0.17   0      0.031
##  2 GWESP (69)               0.917***  2.50  0.11   0      0.006
##  3 time ego                 -0.036    0.965 0.033  0.281 -0.026
##  4 priorVR ego              -0.099    0.906 0.136  0.467  0.042
##  5 priorVR abs. difference  0.183     1.20  0.117  0.117  0.046
##  6 priorAcq ego             0.081     1.08  0.13   0.534  0.038
##  7 priorAcq abs. difference -0.185    0.831 0.124  0.135 -0.002
##  8 groupIdn ego             -0.075    0.927 0.058  0.193  0.016
##  9 groupIdn abs. difference -0.174**  0.841 0.063  0.006  0.002
## 10 same gender              0.139     1.15  0.097  0.152  0.05 
## 11 same ethnicity           -0.086    0.917 0.122  0.478  0.054
```

## Model 3: Dyadic effects

```
siena_res3 <- fit_model(siena_data, siena_eff3)
```

```
## If you use this algorithm object, siena07 will create/use an output file Siena.txt .
## 
## Start phase 0 
## theta: -0.506  0.000  0.000  0.000  0.000  0.000  0.000  0.000  0.000  0.000  0.000  0.000  0.000 
## 
## Start phase 1 
## Phase 1 Iteration 1 Progress: 0%
## Phase 1 Iteration 25 Progress: 0%
## theta: -0.47713  0.56251  0.08465  0.00565 -0.00791  0.01665  0.04756 -0.06157  0.04523 -0.07769 -0.12137 -0.01645 -0.03679 
## 
## Start phase 2.1
## Phase 2 Subphase 1 Iteration 1 Progress: 15%
## Phase 2 Subphase 1 Iteration 2 Progress: 15%
## theta -0.5034  0.8827  0.2732  0.0251 -0.0433 -0.0910  0.0764 -0.1687  0.0520 -0.1268 -0.1642 -0.0160 -0.0557 
## ac  0.0091 -0.2102 -2.6246 -1.3036 -0.2514  0.0537 -0.1162  0.1037  0.2985 -0.1641  0.0846 -0.2594 -0.0149 
## Phase 2 Subphase 1 Iteration 3 Progress: 15%
## Phase 2 Subphase 1 Iteration 4 Progress: 15%
## theta -0.5152  0.8216  0.4622  0.0164 -0.1063 -0.2850  0.1137 -0.2726  0.0163 -0.1579 -0.2070 -0.0169 -0.0961 
## ac -0.15381 -0.37555 -1.33956 -0.90472 -0.59800 -0.06535 -0.30046 -0.04615  0.22522 -0.70452 -0.00931 -0.46296 -0.24387 
## Phase 2 Subphase 1 Iteration 5 Progress: 15%
## Phase 2 Subphase 1 Iteration 6 Progress: 15%
## theta -0.54355  1.24199  0.42319 -0.04141 -0.01441 -0.17193  0.21284 -0.30616 -0.05058 -0.13939 -0.22683 -0.00647 -0.16514 
## ac -0.2763 -0.3058 -0.8103 -0.7334 -0.4341 -0.2516 -0.3769 -0.2838 -0.0768 -0.1903 -0.2256 -0.3754 -0.3503 
## Phase 2 Subphase 1 Iteration 7 Progress: 15%
## Phase 2 Subphase 1 Iteration 8 Progress: 15%
## theta -0.5148  1.1049  0.4468 -0.0399 -0.0520 -0.1189  0.2241 -0.3031 -0.0589 -0.1214 -0.2609  0.0184 -0.1658 
## ac -0.412 -0.401 -0.567 -0.652 -0.573 -0.404 -0.454 -0.386 -0.300 -0.316 -0.349 -0.478 -0.439 
## Phase 2 Subphase 1 Iteration 9 Progress: 15%
## Phase 2 Subphase 1 Iteration 10 Progress: 15%
## theta -0.4921  1.0840  0.3805 -0.0581 -0.0561 -0.0909  0.2273 -0.3545 -0.0850 -0.1398 -0.2654  0.0393 -0.2112 
## ac -0.474 -0.459 -0.529 -0.609 -0.621 -0.464 -0.495 -0.450 -0.407 -0.427 -0.427 -0.498 -0.484 
## theta -0.5013  0.6086  0.4069 -0.0223 -0.0655 -0.2075  0.1427 -0.2252 -0.0316 -0.1000 -0.2007 -0.0118 -0.1220 
## ac -0.509 -0.496 -0.488 -0.587 -0.585 -0.496 -0.516 -0.479 -0.479 -0.503 -0.461 -0.511 -0.521 
## theta: -0.5013  0.6086  0.4069 -0.0223 -0.0655 -0.2075  0.1427 -0.2252 -0.0316 -0.1000 -0.2007 -0.0118 -0.1220 
## 
## Start phase 2.2
## Phase 2 Subphase 2 Iteration 1 Progress: 19%
## Phase 2 Subphase 2 Iteration 2 Progress: 19%
## Phase 2 Subphase 2 Iteration 3 Progress: 19%
## Phase 2 Subphase 2 Iteration 4 Progress: 19%
## Phase 2 Subphase 2 Iteration 5 Progress: 19%
## Phase 2 Subphase 2 Iteration 6 Progress: 19%
## Phase 2 Subphase 2 Iteration 7 Progress: 19%
## Phase 2 Subphase 2 Iteration 8 Progress: 19%
## Phase 2 Subphase 2 Iteration 9 Progress: 19%
## Phase 2 Subphase 2 Iteration 10 Progress: 19%
## theta -0.5802  0.7876  0.3764 -0.0371 -0.0628 -0.1615  0.1486 -0.1782 -0.0363 -0.1122 -0.1907 -0.0249 -0.1547 
## ac -0.942 -0.922 -0.833 -0.593 -0.732 -0.951 -0.963 -0.958 -0.942 -0.716 -0.930 -0.938 -0.965 
## theta: -0.5802  0.7876  0.3764 -0.0371 -0.0628 -0.1615  0.1486 -0.1782 -0.0363 -0.1122 -0.1907 -0.0249 -0.1547 
## 
## Start phase 2.3
## Phase 2 Subphase 3 Iteration 1 Progress: 24%
## Phase 2 Subphase 3 Iteration 2 Progress: 24%
## Phase 2 Subphase 3 Iteration 3 Progress: 24%
## Phase 2 Subphase 3 Iteration 4 Progress: 24%
## Phase 2 Subphase 3 Iteration 5 Progress: 24%
## Phase 2 Subphase 3 Iteration 6 Progress: 24%
## Phase 2 Subphase 3 Iteration 7 Progress: 24%
## Phase 2 Subphase 3 Iteration 8 Progress: 24%
## Phase 2 Subphase 3 Iteration 9 Progress: 24%
## Phase 2 Subphase 3 Iteration 10 Progress: 24%
## Phase 2 Subphase 3 Iteration 100 Progress: 26%
## theta -0.61807  0.81349  0.37225 -0.04413 -0.05735 -0.16589  0.14663 -0.15329 -0.05083 -0.10168 -0.17528 -0.00734 -0.14053 
## ac -0.5868 -0.6722 -0.2570  0.0128 -0.1453 -0.6278 -0.6276 -0.5106 -0.4832 -0.1305 -0.6077 -0.5477 -0.4765 
## theta -0.6246  0.8485  0.3665 -0.0497 -0.0582 -0.1575  0.1511 -0.1698 -0.0465 -0.1007 -0.1737 -0.0151 -0.1591 
## ac -0.5640 -0.6526 -0.3055 -0.0142 -0.1148 -0.6126 -0.6019 -0.4898 -0.4373 -0.1220 -0.5884 -0.4904 -0.4523 
## theta: -0.6246  0.8485  0.3665 -0.0497 -0.0582 -0.1575  0.1511 -0.1698 -0.0465 -0.1007 -0.1737 -0.0151 -0.1591 
## 
## Start phase 2.4
## Phase 2 Subphase 4 Iteration 1 Progress: 30%
## Phase 2 Subphase 4 Iteration 2 Progress: 30%
## Phase 2 Subphase 4 Iteration 3 Progress: 30%
## Phase 2 Subphase 4 Iteration 4 Progress: 30%
## Phase 2 Subphase 4 Iteration 5 Progress: 30%
## Phase 2 Subphase 4 Iteration 6 Progress: 30%
## Phase 2 Subphase 4 Iteration 7 Progress: 30%
## Phase 2 Subphase 4 Iteration 8 Progress: 30%
## Phase 2 Subphase 4 Iteration 9 Progress: 30%
## Phase 2 Subphase 4 Iteration 10 Progress: 30%
## Phase 2 Subphase 4 Iteration 100 Progress: 32%
## theta -0.6425  0.8768  0.3696 -0.0554 -0.0560 -0.1334  0.1474 -0.1521 -0.0394 -0.0935 -0.1799 -0.0195 -0.1403 
## ac -0.2413 -0.4035 -0.2776 -0.0382 -0.0422 -0.2919 -0.1527 -0.0345  0.0534 -0.0187 -0.2805 -0.3252 -0.2444 
## Phase 2 Subphase 4 Iteration 200 Progress: 34%
## theta -0.6352  0.8568  0.3703 -0.0477 -0.0559 -0.1517  0.1547 -0.1724 -0.0580 -0.0917 -0.1756 -0.0224 -0.1500 
## ac -1.81e-01 -2.70e-01 -4.67e-02 -8.96e-05 -8.65e-02 -2.09e-01 -4.64e-02  2.43e-02  1.19e-01 -1.93e-02 -1.81e-01 -2.46e-01 -1.44e-01 
## theta -0.6381  0.8633  0.3678 -0.0546 -0.0571 -0.1553  0.1547 -0.1678 -0.0506 -0.0943 -0.1739 -0.0152 -0.1537 
## ac -0.20202 -0.20802 -0.12033 -0.04225 -0.10797 -0.21971 -0.07191 -0.06043 -0.00359 -0.02868 -0.16604 -0.26607 -0.20649 
## theta: -0.6381  0.8633  0.3678 -0.0546 -0.0571 -0.1553  0.1547 -0.1678 -0.0506 -0.0943 -0.1739 -0.0152 -0.1537 
## 
## Start phase 3 
## Phase 3 Iteration 1 Progress 38%
## Phase 3 Iteration 301 Progress 44%
## Phase 3 Iteration 601 Progress 50%
## Phase 3 Iteration 901 Progress 56%
## Phase 3 Iteration 1201 Progress 63%
## Phase 3 Iteration 1501 Progress 69%
## Phase 3 Iteration 1801 Progress 75%
## Phase 3 Iteration 2101 Progress 81%
## Phase 3 Iteration 2401 Progress 88%
## Phase 3 Iteration 2701 Progress 94%
```

```
tidy_output(siena_res3)
```

```
## # A tibble: 13 × 6
##    parameter                print       exp    SE pvalue  tconv
##    <chr>                    <chr>     <dbl> <dbl>  <dbl>  <dbl>
##  1 degree (density)         -0.638*** 0.528 0.174  0      0.039
##  2 GWESP (69)               0.863***  2.37  0.115  0      0.043
##  3 familiarity              0.368***  1.44  0.068  0     -0.007
##  4 friendship               -0.055    0.947 0.063  0.385 -0.033
##  5 time ego                 -0.057    0.944 0.035  0.098 -0.019
##  6 priorVR ego              -0.155    0.856 0.141  0.272  0.044
##  7 priorVR abs. difference  0.155     1.17  0.111  0.162  0.019
##  8 priorAcq ego             -0.168    0.846 0.149  0.261  0.027
##  9 priorAcq abs. difference -0.051    0.951 0.128  0.693  0.032
## 10 groupIdn ego             -0.094    0.91  0.06   0.119  0.049
## 11 groupIdn abs. difference -0.174**  0.84  0.062  0.005  0.045
## 12 same gender              -0.015    0.985 0.105  0.885  0.002
## 13 same ethnicity           -0.154    0.858 0.124  0.214  0.042
```

# R Environment

```
sessionInfo()
```

```
## R version 4.5.1 (2025-06-13)
## Platform: aarch64-apple-darwin20
## Running under: macOS Tahoe 26.5.1
## 
## Matrix products: default
## BLAS:   /Library/Frameworks/R.framework/Versions/4.5-arm64/Resources/lib/libRblas.0.dylib 
## LAPACK: /Library/Frameworks/R.framework/Versions/4.5-arm64/Resources/lib/libRlapack.dylib;  LAPACK version 3.12.1
## 
## locale:
## [1] en_US.UTF-8/en_US.UTF-8/en_US.UTF-8/C/en_US.UTF-8/en_US.UTF-8
## 
## time zone: America/Los_Angeles
## tzcode source: internal
## 
## attached base packages:
## [1] stats     graphics  grDevices utils     datasets  methods   base     
## 
## other attached packages:
##  [1] RSiena_1.4.7    psych_2.5.3     lubridate_1.9.4 forcats_1.0.0  
##  [5] stringr_1.5.1   dplyr_1.1.4     purrr_1.1.0     readr_2.1.5    
##  [9] tidyr_1.3.1     tibble_3.3.0    ggplot2_3.5.2   tidyverse_2.0.0
## 
## loaded via a namespace (and not attached):
##  [1] sass_0.4.10        utf8_1.2.6         generics_0.1.4     lattice_0.22-7    
##  [5] stringi_1.8.7      hms_1.1.3          digest_0.6.37      magrittr_2.0.3    
##  [9] evaluate_1.0.4     grid_4.5.1         timechange_0.3.0   RColorBrewer_1.1-3
## [13] bookdown_0.43      fastmap_1.2.0      Matrix_1.7-3       jsonlite_2.0.0    
## [17] scales_1.4.0       jquerylib_0.1.4    mnormt_2.1.1       cli_3.6.5         
## [21] rlang_1.2.0        crayon_1.5.3       bit64_4.6.0-1      withr_3.0.2       
## [25] cachem_1.1.0       yaml_2.3.10        tools_4.5.1        parallel_4.5.1    
## [29] tzdb_0.5.0         vctrs_0.6.5        R6_2.6.1           lifecycle_1.0.4   
## [33] bit_4.6.0          MASS_7.3-65        vroom_1.6.5        pkgconfig_2.0.3   
## [37] pillar_1.11.0      bslib_0.9.0        gtable_0.3.6       glue_1.8.0        
## [41] rmdformats_1.0.4   xfun_0.52          tidyselect_1.2.1   rstudioapi_0.17.1 
## [45] knitr_1.50         dichromat_2.0-0.1  farver_2.1.2       nlme_3.1-168      
## [49] htmltools_0.5.8.1  labeling_0.4.3     rmarkdown_2.29     compiler_4.5.1
```
